# Assessing the quality of amoxicillin in the private market in Indonesia: a cross-sectional survey exploring product variety, market volume and price factors

**DOI:** 10.1136/bmjopen-2024-093785

**Published:** 2025-07-22

**Authors:** Amalia Hasnida, Mawadatti Rahmi, Ayu Rahmawati, Yusi Anggriani, Frank C M van Leth, Maarten Olivier Kok

**Affiliations:** 1 Health Care Governance, Erasmus University Rotterdam, Rotterdam, The Netherlands; 2 Center for Pharmaceutical Policy, Management, and Services Studies, Faculty of Pharmacy, Universitas Pancasila, Jakarta, Indonesia; 3 Health Sciences, Vrije Universiteit Amsterdam, Amsterdam, The Netherlands; 4 Amsterdam Public Health Research Institute, Amsterdam, The Netherlands

**Keywords:** Cross-Sectional Studies, EPIDEMIOLOGY, Health policy, Public health, PUBLIC HEALTH, Quality in health care

## Abstract

**Abstract:**

**Objectives:**

To assess the quality of amoxicillin products in Indonesia’s private market by surveying the range of products available across different areas, followed by product sampling and laboratory testing.

**Design:**

A cross-sectional survey employing mystery shoppers to purposively sample the widest possible range of amoxicillin products available to patients across different areas in Indonesia.

**Setting:**

Licensed and unlicensed medicine outlets in remote, rural and urban areas and online.

**Participants:**

Amoxicillin products that are sold to patients as oral solid and dry liquid formulations.

**Main outcome measures:**

Quality of amoxicillin products, assessed using compendial testing of active pharmaceutical ingredient content and dissolution. Samples that failed any quality test were classified as substandard or out-of-specification (OOS). The raw prevalence of substandard amoxicillin was adjusted based on the national market volume of each product variant.

**Results:**

We surveyed 476 outlets, mostly pharmacies (68.5%), websites (19.7%) and drug stores (10.9%). Among the 120 collected samples, there were 59 distinct products, collectively representing 95% of the estimated market volume for oral solid products and 65% for dry syrups. 12 out of 110 oral solid samples tested OOS (10.9%), as did 3 out of 10 dry syrups (30%). The samples that failed originated from various areas and types of outlets. We found no relation between the price and quality of amoxicillin.

**Conclusions:**

The oral solid amoxicillin products that tested OOS represent an estimated 12.7% of the national market volume. We found no relation between the price and quality of amoxicillin. Combining product-variety sampling with data on market volume presents a promising approach to gain insight into the prevalence of poor-quality medical products using a relatively small sample size.

**STRENGTHS AND LIMITATIONS OF THIS STUDY**

- A key strength is the comprehensive survey of 476 private outlets across 4 areas in Indonesia, including licensed, unlicensed and online vendors, providing a robust picture of the marketplace landscape.
- Diverse sample comprising 59 distinct amoxicillin products, representing 95% of the estimated national market volume for oral solid formulations.
- A limitation of this study is that, although the 120 collected samples cover a large share of the Indonesian market, several authorised oral solid products representing 5% of the market volume were not included.

## Introduction

 Substandard and falsified medicines pose a threat to patient health, lead to waste of resources, undermine confidence in health systems and contribute to antimicrobial resistance.^1^ Substandard, often referred to as ‘out-of-specification (OOS)’, denotes an authorised product that fails to meet either its quality standards, specifications or both. Falsified medicines deliberately or fraudulently misrepresent their identity, composition or source.^2^ Despite an increased focus on medicine quality prompted by alarming incidents,^3^,^5^ robust evidence about the prevalence of poor-quality medicines remains scarce.^6 7^

Medicine regulators are tasked with assuring the quality, safety and efficacy of medicines on the market by routinely sampling and testing products that are available to patients.^8^ It is essential that regulators sample medicines at the point where patients get them, as poor storage and distribution practices may lead to the degradation of medicines along the supply chain,^9^ and outlets may also sell expired, unauthorised and falsified products.^10^

Regulators face many challenges in conducting effective postmarket surveillance. They have to choose which products are most at risk, collect them from the market, and conduct quality tests.^11^ In most countries, thousands of medications have received market authorisation. For most medicines, there are multiple product variations on the market. For example, amoxicillin is available in diverse forms (tablets, capsules, dry syrup) and varying strengths (250 mg, 500 mg, 1000 mg). Multiple companies manufacture amoxicillin, with some marketing it under distinct brand names, and others offering an unbranded generic option.

After a regulator decides to sample a specific product variation, the subsequent operational challenge is sampling that product in the market.^12^ The sales volume and distribution of product varieties can vary significantly. Some products have large market volumes and are sold across the country, while others may be sold in small volumes and available only in specific areas.

Since medicines may degrade along the supply chain,^9^ regulators also need to choose from which location, market segment and type of outlets they will sample.^12^ A private pharmacy in a bustling metropolis, a small clinic in a remote village or an outlet that is not licensed to sell prescription-only medicines, which could be a drug store, market vendor, health workers or a website.^13 14^ Testing the quality of medicine in the laboratory is also not straightforward, as there are different testing parameters and quality standards. Meanwhile, many regulators struggle to perform their routine tasks due to the high cost of testing and insufficient human, financial and physical resources and a lack of enforcement capacity.^8 15 16^

The different factors that make postmarket surveillance challenging are all present in Indonesia. Its 278 million citizens^17^ are spread across 7000 inhabited islands.^18^ The pharmaceutical market is huge. There are over 19 000 registered pharmaceutical products,^19^ produced by 225 manufacturers, of which 88% are domestic companies.^20^ Most manufacturers are located on Java Island in the centre of the country,^21^ from which there are complex supply chains to the outer islands of the archipelago, which can be thousands of kilometres away.^22 23^ Medicines are provided to patients by roughly 10 100 primary health centres, 3100 hospitals and sold in 22 000 authorised retail pharmacies.^24 25^ In addition, there are 10 800 registered drug stores that are allowed to dispense over-the-counter medicines, but often also sell prescription-only medicine, such as antibiotics.^25 26^

The vibrant private sector plays a large role in the direct provision of medicines to patients. While most Indonesians have social health insurance and are entitled to free essential medicines from public facilities, 79% of expenditures on medicines is still paid out-of-pocket to private outlets.^27^ Patients are driven to private outlets because public facilities are out of stock^28^ or difficult to reach^29^ or overburdened, leading to longer waiting times.^30^ Patients also choose to buy medicines from private outlets because they believe the more expensive branded medicines are of higher quality.^31 32^

Despite the widely documented incidents—such as falsified vaccines in 2016 and contaminated cough syrup in 2022^33 34^—there are very few studies assessing the quality of medicines available to patients in Indonesia, particularly in the private sector.^35 36^ In this study, we focus on amoxicillin trihydrate, the most widely used antibiotic in outpatient settings,^37^ including in Indonesia.^38^ As in many countries, hundreds of amoxicillin products are authorised for the Indonesian market.^19^ Ensuring their quality is crucial, as substandard antibiotics not only pose risks to patients’ health but can also contribute to the escalation of antimicrobial resistance.^39^

To our knowledge, there are no studies that purposively examine the variety of products of a specific antibiotic that are available to patients within a country, taking into account brand variations, market volume and type of outlets, to test their quality.

The aim of this study is to assess the quality of amoxicillin within the Indonesian private market by examining the diversity of products sold across different areas and various types of medicine outlets. Additionally, we analysed the market volume of the tested products and assessed the relationship between the price and quality of amoxicillin.

We focused on the private sector, as this is where the majority of patients obtain their medicines, and we expected to encounter the widest variety of available products. In four different areas and the online market, we surveyed the range of products available and purposively sampled the widest possible variety of amoxicillin products. Subsequently, we conducted pharmaceutical analysis to evaluate their quality. This included assay testing to quantify the percentage of active ingredients and dissolution testing to determine if the products released the active substance in a timely manner.^40^,^42^

## Methods

Methods are described following the Medicine Quality Assessment Reporting Guideline^43^ (online supplemental file 1). Our study design is a cross-sectional survey of product varieties, prices and pharmaceutical quality testing analysis.

### Study medicine

Our study focused on amoxicillin, considering its public health importance, as it is among the antibiotics with the highest utilisation in Indonesia.^38^ Poor-quality amoxicillin might also trigger antimicrobial resistance,^44^ a true concern in the country.^45^ In addition, many amoxicillin products are registered with different price points,^46 47^ enabling us to examine the product variety and medicine prices and assess their quality. We discussed our study design with the Indonesian medicine regulators,^48^ who suggested including amoxicillin based on their previous inspection records.

### Samples definition

We defined a single sample as one finished pharmaceutical product (with amoxicillin as active pharmaceutical ingredient, API), of one dosage (strength and form), of one brand, from one manufacturer, and collected at one location, at one time).^49^ We classified medicine types as branded generics and unbranded, International Nonproprietary Name generics.

### Sampling areas and types of outlets

Samples were collected between November 2020 and November 2021 in four geographical areas and online (online supplemental file 2). Based on the initial assumption that the long chain of medicine supply might introduce degradation risks,^12^ we purposively selected four different areas: remote (East Nusa Tenggara province, NTT), semirural (Malang district in East Java province) and two urban areas of the Greater Jakarta region (Jakarta and the satellite city Bekasi). In the remote and semirural districts, we visited one location with a larger population (eg, provincial capital) and one with a smaller size (eg, district or administrative city).

Our sampling encompassed both licensed outlets, such as pharmacies, and outlets not authorised to sell prescription-only medicines, including drug stores, health providers and online platforms. The distribution of outlets varied across sampling areas, determined by outlet availability in each area and the feasibility of on-site visits. We located these outlets by cross-referencing medicine outlet directories from the subnational health authorities with empirical location mapping from previous surveys^26 35^ and by conducting product searches on various Indonesian online marketplace platforms. In the Greater Jakarta area, our sampling of drug stores focused on two concentrated medicine trade areas.^50^ For online sources, we specifically selected unlicensed vendors without permission to trade prescription-only medicines.

### Secondary data collection and analysis

To prepare for sampling, we started by creating a sampling plan with an overview of the different amoxicillin products in the Indonesian market and the market volume for each distinct product. We combined data about registered medicines from the public regulatory database (National Medicines Regulatory of Indonesia, 2016–2018) and the products listed in public procurement data (National Procurement Agency, 2018) and obtained market data from IQVIA, a pharmaceutical data services firm (IQVIA MIDAS ® Quarterly Sales Data from 2020 which were obtained under license from IQVIA and reflect estimates of marketplace activity). These MIDAS data estimate market sales volumes and list prices of most amoxicillin products in the Indonesian market. We used the Indonesian Total Market Audit data, which estimates product supply by combining the actual sales from pharmaceutical companies with sales projections from retail channels such as pharmacies, drug stores and hospitals.

### Amoxicillin forms

We defined form as the way a medicine is presented.^51^ We chose to focus on oral solid products (tablets and capsules) as these have the highest estimated market volume in the Indonesian market. In the final stage of the data collection, an expert from the WHO suggested including dry syrup as well. We collected 10 dry syrup samples, only in the Greater Jakarta area.

#### Sampling strategy

We aimed to collect a total of 120 samples. Initially, we planned to target preselected amoxicillin products that had specific risk factors for being substandard, based on our previous assessments,^6^ and collect an equal number of samples in each area. In the first area, we learnt that the range of products available from outlets was very limited. We, therefore, decided to focus our sampling strategy on purposively collecting the largest possible variety of amoxicillin products available on the market, which we will refer to as product variety sampling. The total sample size (N=120) is based on the maximum feasible budget for sampling and pharmaceutical analysis. We expect this sample size to cover around 90% of the estimated market share.

Prior to sampling, the data collectors surveyed product variety by recording all brands and dosage forms sold in a particular outlet. In each new area, we focused on trying to sample new product varieties. In case a product was available from a different type of outlet, we would sample it again (eg, brand X in area 1 from a pharmacy, and brand X in area 2 from an unlicensed drug store). For dry syrup, we collected each different brand for each sample based on the availability at the outlet (N=10).

#### Product survey and sample collection

In each area, we recruited local enumerators with experience in health research (17 in total). We trained them for 2 days on the purchasing methods using vignettes, samples handling, storage and delivery. We also prepared medical prescriptions to collect the samples as necessary.

On arrival at the sampling outlet, data collectors asked which amoxicillin products were sold, which they recorded on their mobile phones. If asked regarding specific symptoms by the pharmacists or storekeepers, they responded by presenting a case of acute sore throat that persisted for more than 3 days, either for themselves or another family member (for their child in the case of dry syrup samples). Only on request, they provided the prescriptions.

To select which products to sample, the enumerators referred to a daily sampling plan of targeted product varieties yet to be collected.

To meet the technical requirements of pharmaceutical analysis, for each sample the enumerators aimed to purchase 40 tablets or capsules (usually in 4 foil strips or blisters) or 5 bottles of dry syrup. When there were no new variations available at the outlet, or the quantity of the available product was less than the minimum units required, the enumerators left the outlet without any purchase.

The enumerators recorded the collected samples data on an open-source mobile application called KoBo Collect.^52^ Data about the details of the outlet and sampling location were recorded on site. Product details were completed in the field office (forms are available in online supplemental file 3). Data collectors visually inspected the samples using a magnifying glass to inspect any damaged packaging.

### Sample handling and storage

We labelled each sample with a unique barcode. Each sample was stored in a zipper bag labelled with a barcode, the date of data collection and sampling location. We stored the samples in tight containers before pharmaceutical analysis following the procedure of proper amoxicillin storage at controlled room temperature.^53^ We included a temperature data logger in the container. The identity, quantity and expiry dates of the samples were verified again by the laboratory personnel.

#### Pharmaceutical analysis

Samples were tested at PT Equilab International in Jakarta, a private laboratory certified with ISO/IEC 17025, referring to the US Pharmacopeia (USP) 42 National Formulary 37^53^ from December 2020 to January 2022 on average 93 days (17–331 days) after sample collection. The laboratory validated all methods for all dosage forms before testing (detailed testing protocol is in online supplemental file 4).

The laboratory staff performed visual inspections by examining the physical attributes of each sample, for example, colour and shapes of the tablets or bottles of the dry syrups. We conducted assay testing to quantify the percentage of active ingredients and dissolution testing to determine if the products released the active substance in a timely manner.^40^,^42^

Assay and dissolution were performed using HPLC-UV (Waters Alliance 2695, with UV Detector 2489), Spectrophotometer (Shimadzu, UV-1800) and Dissolution Tester with USP Paddle All-Teflon 15” Long. Per protocol, the staff handling the packaged samples was different from the staff conducting the tests. All samples (N=120) were tested in terms of simple visual inspection and assay. Dissolution was only performed on solid dosage forms (tablets and capsules).

The laboratory staff used the sample barcodes throughout the testing procedure. The research team and a quality assurance adviser reviewed the raw measurement data and certificates of analysis. The quality testing parameters and compliance standards are summarised in online supplemental file 5. Q is the targeted amount of active ingredient, expressed as a percentage of the labelled content of the dosage unit, which should be dissolved within a certain time.^41^

Following USP42 NF37, we considered a sample as OOS if it falls into any of these following categories:

- Assay results are outside the stated limits.
- Dissolution stage 1 result shows each unit is lower than Q+5%.
- Dissolution stage 2 average result of 12 units is <Q, and at least a unit <Q–15%.
- More than 2 units are <Q–15%, and at least a unit is <Q–25%.

Given the limited number of tablets, capsules or bottles of each sample that can be collected using mystery shopping, we performed dissolution stages 1 and 2.^41^ The assay protocol was adjusted and validated using two units of tablets instead of five as stated in USP 42 NF 37. This testing protocol was designed in consultation with experts from USP and in line with a review indicating a minimal likelihood of atypical performance in stage 3 for products that pass stages 1 and 2.^54^

### Medicine prices and quality assessments

When purchasing the samples on-site, the enumerators recorded the price they paid in the respective outlets on the KoBo collect, later referred to as the patient purchase price. Price was analysed in the smallest counting units, for example, per capsule or tablet for oral solid dosage forms and 5 mL for dry syrups. As we captured the actual price paid in the market, no reference prices were used in this study.

### Data management and analysis

Data were analysed using STATA MP V.18. For five oral solid products, market volume data were unavailable. To address this, we performed imputation using the median of all sampled oral solid products.

To get an indication of the prevalence of poor-quality amoxicillin in the private market, we weighted the results by the sales volume of each product. For each product, we multiplied the percentage of samples that failed a quality test by the market volume of that product. To examine the relationship between price and quality, we conducted logistic regression. The independent variable was the price paid by patients (selling price, which we calculated per standard counting units for tablets and dry syrup) sourced from our empirical mystery shopping survey, and the dependent variable was the outcome of pharmaceutical analysis, indicating whether the product was within or outside of specification. We also conducted logistic regression to study the relationship between the estimated market volume for oral solid products (independent variable) and at least the one-time failure of pharmaceutical analysis (dependent variable).

### Patient and public involvement

We did not involve patients in this study. We consulted the study design with the Indonesian National Medicine Regulatory Authority in a series of meetings prior to the empirical data collection between July and October 2019.

## Results

### Amoxicillin product variety in the Indonesian market

At the beginning of 2020, the Indonesian regulator had authorised 185 amoxicillin products for the market, including 104 different oral solid products (tablets or capsules) and 81 different dry syrup products. The oral solid products were available in 250, 500 and 1000 mg tablets and capsules, while dry syrups were available as 125 and 250 mg/5 mL.

Pharmaceutical market data provided the sales volume estimates for 174 products (95 oral solid and 79 dry syrup) (illustrated in online supplemental file 6). The estimated market volume of the oral solid products was 359 million doses and 97 million doses of dry syrups. The market leader was accounting for 24.3% of the volume of oral solid products and 7.1% (dry syrup) of the total market share. Branded generics products dominated both dosage forms (66 out of 95 oral solid and 59 out of 79 dry syrups).

### Visited outlets, product variety identified and sampled

In total, we visited 476 outlets in the four sampling areas and online (details are in online supplemental file 7). We started our data collection in the remote NTT area, where the availability of drug stores was notably limited. Through visits to 47 outlets, we gathered 14 samples encompassing seven distinct amoxicillin product varieties. In the second area (East Java), we visited 132 outlets, identified 38 different products, most of which were not present in the first sampling area. We gathered 34 samples, including 27 distinct products. In the third area (Jakarta), we visited 144 outlets, including 43 drug stores, from which we accumulated 29 samples, introducing 10 new product variations into our dataset. Moving on to Bekasi, a satellite city of Jakarta, we identified a total of 31 distinct products and bought 18 samples. As a final step, we conducted targeted online searches to buy any additional product varieties available. Through online sources, we pinpointed an additional nine new products and bought seven of them.

In total, we identified 57 different oral solid products in the four sampling areas and online and collected 49 different product varieties. Eight products were not sampled because they were not sold in sufficient quantities for pharmaceutical analysis (minimum 20 tablets) or online vendors did not respond to our attempts to order products.

### Description of collected samples

We collected samples as finished pharmaceutical products. In total, we collected 110 oral solid samples in the four sampling areas and online as summarised in table 1. This encompasses 59 different varieties of amoxicillin products, and in several cases, multiple samples of the same product variety, gathered from different types of outlets. In the fourth sampling area, we also collected 10 dry syrup samples, leading to a total of 120 samples. Most samples (93.3%) were obtained without medical prescriptions, and almost all samples (99.2%) were manufactured in Indonesia. We had no information about the source of APIs in each sample. We collected one sample from an online vendor that had no information about the country of origin. In accordance with an agreement with the Indonesian medicine regulator, we are not able to report the brands or manufacturers of the tested samples.

**Table 1:** Descriptions of collected samples

Categories	Frequency	Distinct products*
Total collected samples	120	59
Products characteristics as labelled		
Un-branded/INN generic products	28 (23.3%)	12 (20.3%)
Branded generic products	92 (76.7%)	47 (79.7%)
Dosage forms		
Capsule 250 mg	7 (5.8%)	2 (3.4%)
Capsule 500 mg	18 (15%)	5 (8.5%)
Tablet 500 mg	83 (69.2%)	40 (67.8%)
Tablet 1000 mg	2 (1.7%)	2 (3.4%)
Dry syrup 250 mg/5 mL	2 (1.7%)	2 (3.4%)
Dry syrup 125 mg/5 mL	8 (6.7%)	8 (13.6%)
Packaging		
Strip	109 (90.8%)	48 (81.4%)
Blister	1 (0.83%)	1 (0.83%)
Bottle	10 (8.3%)	10 (8.3%)
Other characteristics†		
Split batch (>1 batch numbers)	9 (7.5%)	N/A
Damaged packaging	2 (1.7%)	N/A
Obtained from air-conditioned outlets	22 (21%)	N/A
Collected without prescriptions	112 (93.3%)	
Manufacturing		
Domestic producer (Indonesia)	119 (99.2%)	58 (98.3%)
Origin unknown	1 (0.8%)	1 (1.7%)

* Distinct product: one amoxicillin brand, of one dosage, in tablets or capsules or dry syrups, and from one manufacturer.

† Distinct products are not applicable for these categories since one product can have multiple versions of characteristics.

INN, International Nonproprietary Name; N/A, not available.

### Pharmacopeial testing results

All samples were identified as amoxicillin and no samples were entirely depleted of amoxicillin. The visual inspection reported no crushed solid forms in their primary packaging. There was one dry syrup sample with suspension powder which leaked in a bag separate from the primary packaging (bottle).

**Figure 1:**
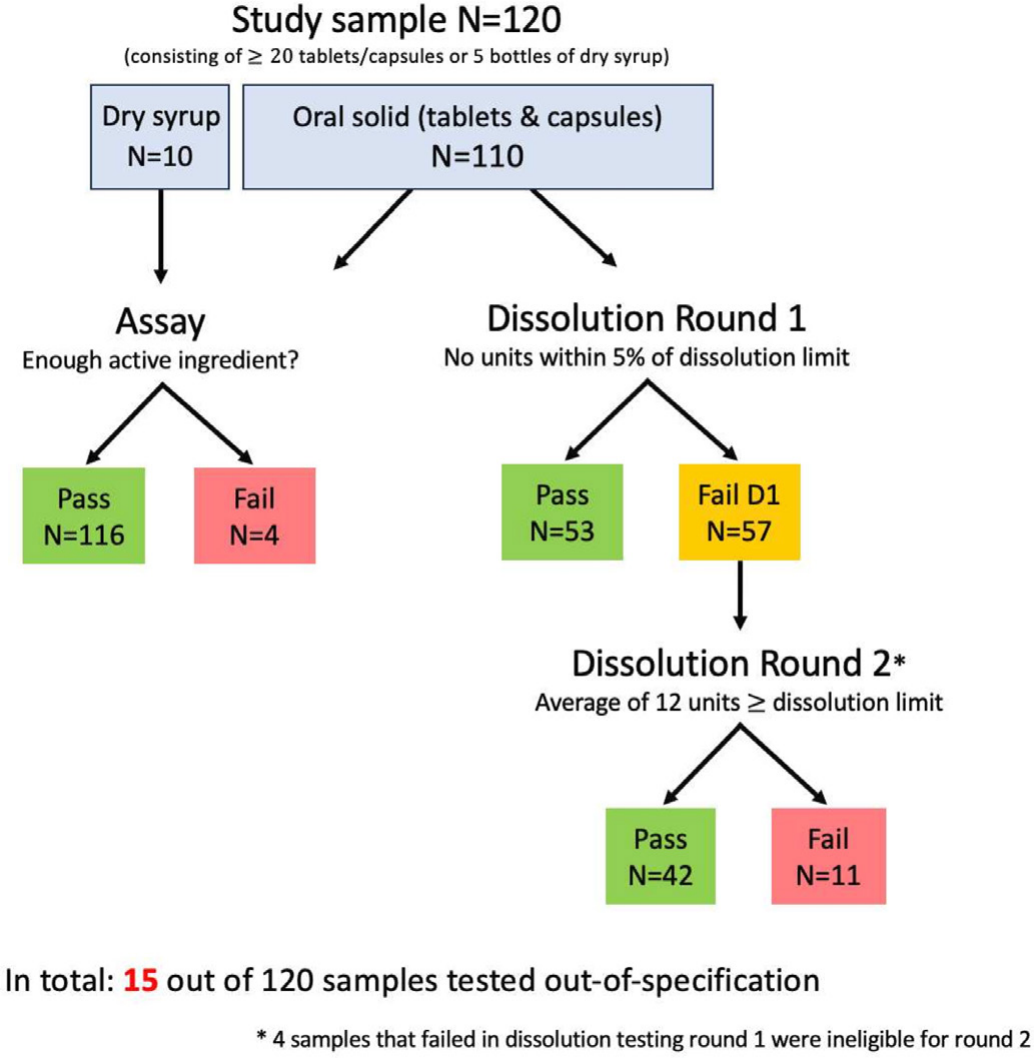
Flow chart of pharmaceutical analysis and results.

Figure 1 provides a flow chart of the assay and dissolution testing and an overview of the results. Four out of 120 samples tested OOS on assay, three of which were dry syrups. All 110 oral solid samples were tested in stage 1 dissolution testing. 57 samples failed in stage 1, but we only included 53 in stage 2 testing. Four samples had to be excluded for the second round, as insufficient tablets or capsules were available (N=1) or samples beyond their expiry date (N=3). Of the 53 samples that were tested in dissolution stage 2, 11 samples were OOS. In total, we report that 15 out of 120 samples were OOS (12.5%). The OOS samples were tested on average of 700 days (113–1026 days) before their expiry date for assay and 587 days (−1 to 920 days) for dissolution stage 2.

The OOS samples in assay consist of the following dosage forms: tablets 500 mg, dry syrup 250 mg/5 mL and dry syrup 125 mg/5 mL (N=2). The dosage forms of the 11 samples that were OOS in dissolution testing were: 250 mg capsule (N=1), 500 mg capsule (N=1), 500 mg tablets (N=9). The sample without information on manufacturing country or market authorisation number did meet both the assay and dissolution specifications criteria. The dry syrup sample with leaked powder failed in assay with the lowest percentage of labelled amoxicillin (72.8%).

We found OOS samples in both licensed and unlicensed outlets as summarised in table 2. We only collected dry syrup samples in Jakarta and Bekasi. We sampled the most from pharmacies where we found the highest numbers of OOS samples (12.1% for oral solid and 60% for dry syrup). Out of two OOS oral solid samples purchased from online vendors, one tablet has the lowest dissolution percentage (45.2%, well below the minimum of 75%). None of the samples that failed a laboratory test came from the most remote area (NTT). Bekasi had the highest proportion of OOS samples both for oral solids (16.7%) and dry syrups (50%). One of the fifteen samples that tested OOS came from a split batch.

**Table 2:** Proportion of quality testing outcomes by sampling areas and types of outlets

Oral solid dosage forms			
By sampling areas			
Areas	Total samples collected (N=110)	Meet specification	Out-of-specification (OOS)
Jakarta	29	25 (86.2%)	4 (13.8%)
East Java	34	31 (91.2%)	3 (8.8%)
Bekasi	18	15 (83.3%)	3 (16.7%)
Online	15	13 (86.7%)	2 (13.3%)
East Nusa Tenggara	14	14 (100%)	0 (0%)

By types of outlets			
Outlets	Total samples collected (N=110)	Meet specification	OOS
Pharmacies	66	58 (87.9%)	8 (12.1%)
Drug stores*	25	23 (92%)	2 (8%)
Health providers (ie, physicians and midwives)*	4	4 (100%)	0 (0%)
Online vendors*	15	13 (86.7%)	2 (13.3%)

Dry syrup dosage forms			
By sampling areas			
Areas	Total samples collected (N=10)	Meet specification	OOS
Jakarta	6	5 (83.3%)	1 (16.7%)
Bekasi	4	2 (50%)	2 (50%)

By types of outlets			
Outlets	Total samples collected (N=10)	Meet specification	OOS
Pharmacies	5	2 (40%)	3 (60%)
Drug stores*	5	5 (100%)	0 (0%)

* Unlicensed outlets to sell antibiotics.

**Figure 2:**
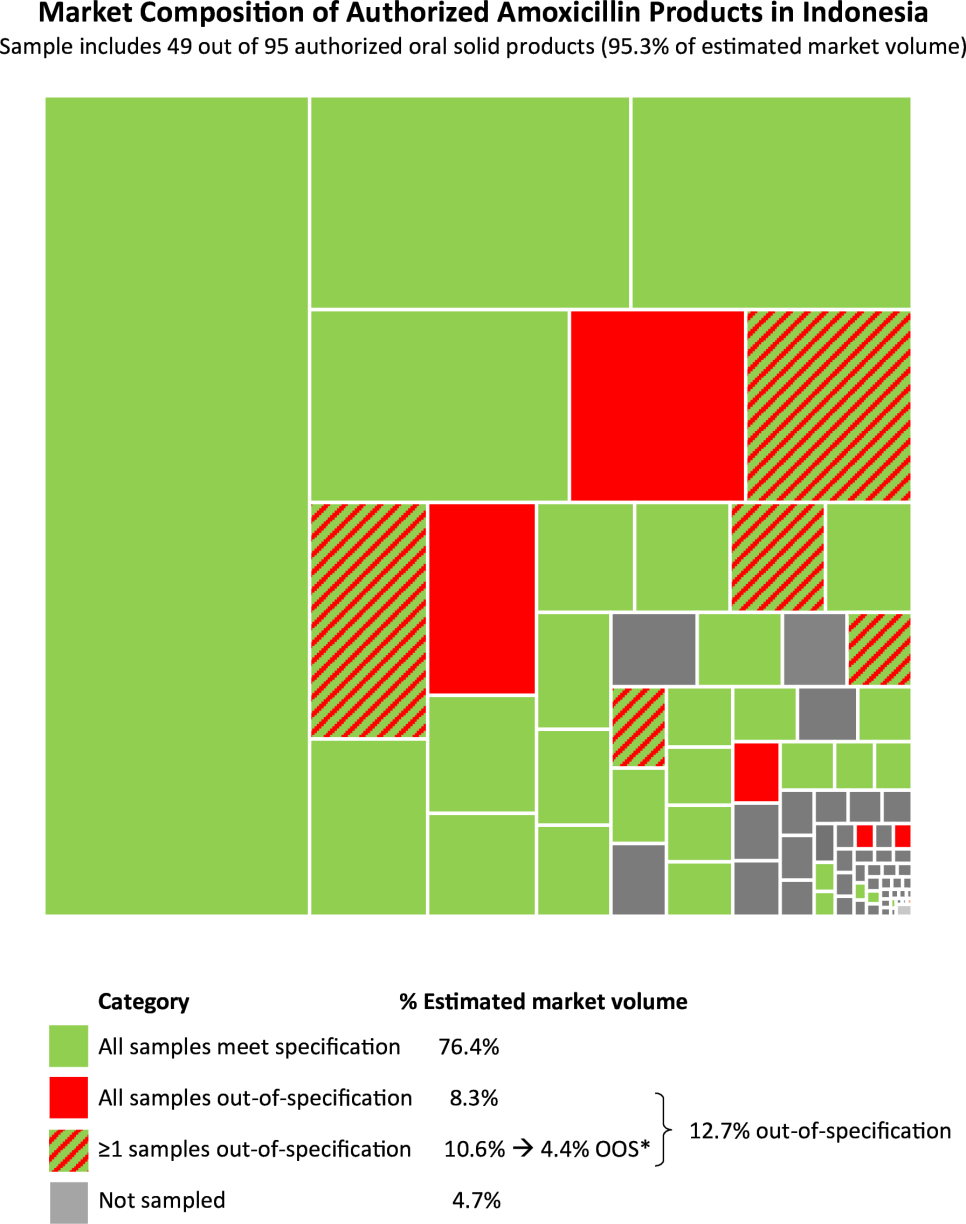
Estimated market volume of amoxicillin oral solid products (N=95) sold in the Indonesian market from October 2019 to September 2020; mapped to sampled and tested products. *After weighting for % of sampled product that tested OOS. Source: This is based on internal analysis by Hasnida *et al* 2025 using data from the following source: IQVIA MIDAS Quarterly Sales for the period (October 2019–September 2020) reflecting estimates of real-world activity. Copyright IQVIA. All rights reserved. OOS, out-of-specification.

Figure 2 provides the estimated share of market volume of the sampled amoxicillin products and the volume of the products that tested OOS in pharmaceutical analysis. Figure 2 shows that the 49 distinct amoxicillin products that we sampled represent 95.3% of the estimated market volume for oral solid products. There were five distinct products for which at least one sample tested within specification and at least one sample tested OOS.

After imputing the estimated market volume of one OOS product (2.4 million) and weighting the frequencies of testing failures (per product), we calculated the estimated total volume of OOS oral solid products in the private market to exceed 46 million, constituting 12.7% of the total private market. We found no relation between the estimated market volume of oral solid products and quality outcomes (OOS on assay and dissolution) (OR=1, 95% CI 0.99 to 1.00).

Online supplemental file 8 provides the estimated share of market volume of the 10 dry syrup products that we sampled. Two OOS products in assay (N=2) had low market volume and one had no market volume data; hence, these are not indicated in this figure.

### Medicine prices and quality outcomes

There were large variations in the patients purchase price of amoxicillin. Calculated per tablet or capsule, the cheapest amoxicillin product of 250 mg cost IDR 400, while the most expensive cost IDR2525. Large price variations were also found for 500 mg (IDR320–IDR6500) and 1000 mg (IDR7000–IDR14 286). For dry syrup, the price also varied significantly (per 5 mL) for both 125 mg (IDR292–IDR1917) and 250 mg (IDR458–IDR833).

**Figure 3:**
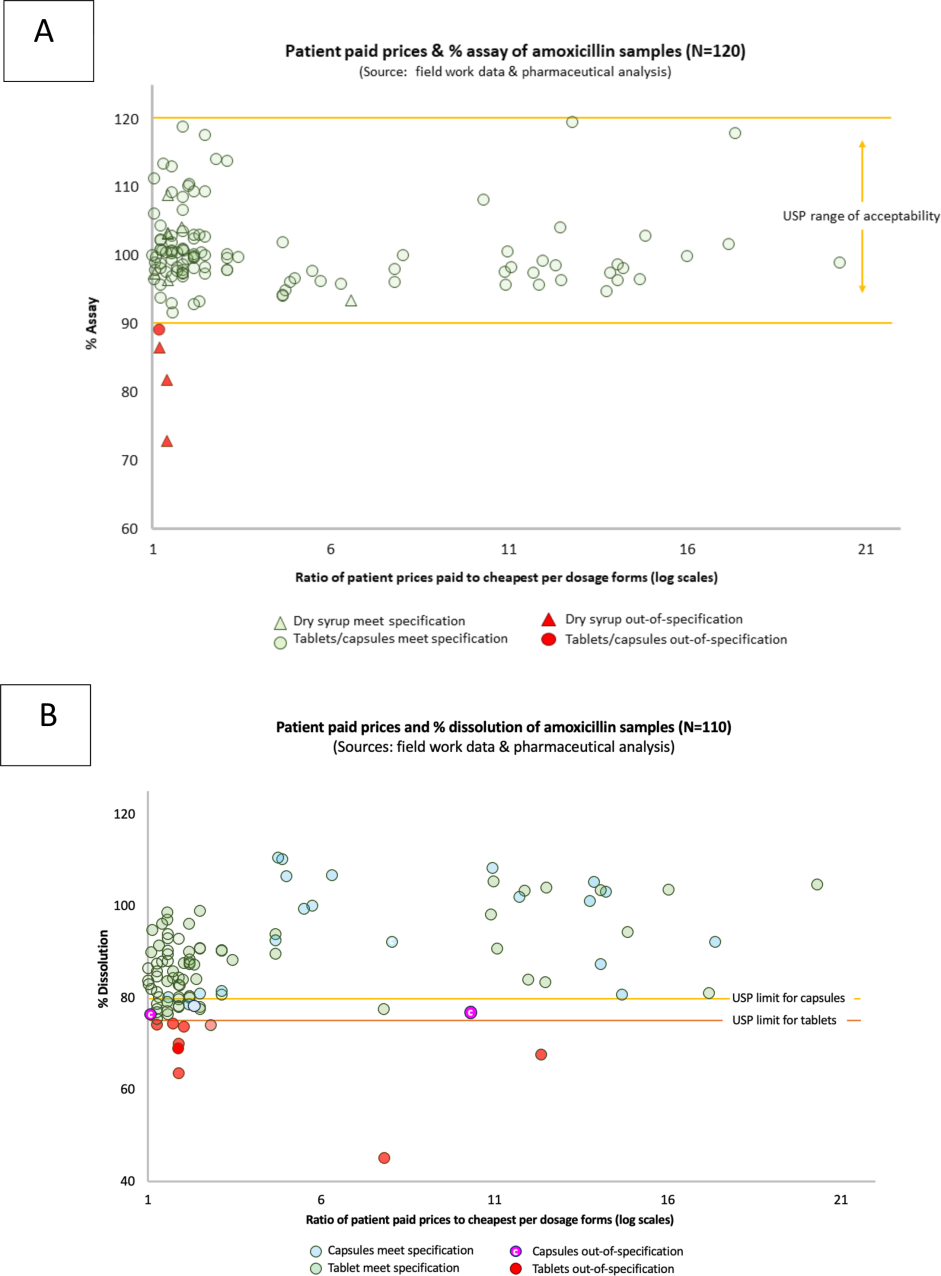
Scattered plots of ratio of patient paid prices to cheapest per dosage forms and percentage of assay testing results (N=120) (**A**) or dissolution (N=110) (**B**). USP, US Pharmacopeia.

Figure 3 provides two scattered plots of ratio for each medicine price to cheapest per dosage forms in log scales for assay and dissolution test, respectively. We found no relation between the price of amoxicillin and quality outcomes (OOS on assay or dissolution) (OR=1, 95% CI 0.99 to 1.00).

## Discussion

Our study assesses the quality of amoxicillin available in the Indonesian private market by examining a large variety of products sold in different areas and from different types of outlets. We surveyed 476 outlets and collected 120 samples that include 59 different products. These 59 products make up 95.3% of the estimated market volume for oral solid products and 65.2% for dry syrups. 15 out of 120 samples (12.5%) tested OOS, of which 4 samples failed in assay and 11 in dissolution. The samples that failed came from different areas and types of outlets. We found no relation between the price of amoxicillin and the results of quality testing. We did not identify any samples indicative of falsified medicines (eg, those completely lacking amoxicillin or containing an alternative API).

The percentage of samples testing OOS (N=15/120) needs to be carefully interpreted. Most failed samples were within 10 percentage points of the allowed limits for assay or dissolution. While the threat to individual patients may be confined to only the lowest-scoring products, the danger posed by substandard antibiotics is substantial.^39^

Insufficient amounts of APIs or poor dissolution features could contribute to the development of antimicrobial resistance,^44^ a significant concern given the hundreds of millions of amoxicillin doses consumed annually in Indonesia. Amoxicillin is frequently identified as a poor-quality antibiotic in surveys in other countries.^55^ Comparing our results to data from the Indonesian medicine regulator is not possible. While the regulator implements a proactive postmarket surveillance programme, it does not release disaggregated results of quality per medicine.^56^

A particular concern is that 3 out of 10 dry syrup samples tested OOS in the assay. Dry syrup amoxicillin is commonly prescribed for paediatric patients who face challenges in swallowing tablets or capsules. Its liquid form allows for easier administration, making it a suitable choice for young patients who require accurate doses. Prior studies have predominantly focused on the quality of tablets and capsules, with limited attention to dry syrup formulations.^57^,^59^ We urgently recommend a more detailed examination of the quality compliance parameters for dry syrup formulations, particularly in larger sample sizes. Additionally, more research with experts such as pharmaceutical scientists and chemists is paramount to understanding the technical factors behind the quality testing results of amoxicillin in liquid formulations,^60^ including dry syrups or adding more evidence on dissolution testing in general.^61 62^

In terms of methodology, our sampling strategy, which focused on collecting the broadest variety of products available to patients from private outlets, offers insights into distribution variability. Previous studies assessed the quality of various medicines in Indonesia but did not explicitly focus on product variability in their sampling strategies.^35 36 63 64^ Amoxicillin products were not evenly distributed across the four sampling areas and the online market. In our initial and most remote sampling area, we found a very limited variety of available products. In the subsequent areas, we found a much larger variety of products. Despite an open market system, it is worth noting that almost all of the samples collected were manufactured locally, indicating that Indonesia has a large domestic pharmaceutical production to meet national demand.^20^

The combination of product-variety sampling and data about the market volume of distinct products enables the use of a relatively small and focused sample (110 oral solid samples, including 49 out of 95 authorised oral solid products) to provide an indication of the quality of a substantial portion of the estimated market volume (95.3%). The 12 oral solid samples that tested OOS account for an estimated 12.7% of the market volume, equivalent to over 46 million doses.

An advantage of our sampling strategy is its focus on the most informative samples, a crucial consideration given the expense of testing the quality of medicines.^65^ However, as demonstrated in our results, it is noteworthy that multiple samples of the same product could yield different quality testing results. This underscores the importance of further investigation into the factors influencing product quality, including batch-to-batch variation and product degradation in the field.

One of the challenges in assuring medicine quality is that poor handling, storage and distribution practices along the supply chain can lead to product degradation. We anticipated that, due to lengthy and vulnerable supply chains, products sold in more remote areas and unlicensed outlets were more likely to test OOS.^12^ Our results do not support this hypothesis. While we observed notable variation in the results of quality testing between areas and types of outlets, our sampling strategy focused on product variety and was not designed to compare differences between areas and outlet types.

The proportion of OOS samples recorded in this study should not be interpreted as a prevalence estimate of all substandard amoxicillin across Indonesia. Our study specifically focused on tablets and capsules available to patients in private outlets, representing an estimated market volume of approximately 340 million doses per year. While public sector facilities are supplied by the same manufacturers, they may source from a smaller number of them, making it challenging to generalise our findings.^36^

Our study illustrates the potential of combining product-variety sampling with market volume data, indicating how a relatively small and focused sample can provide insight into a significant portion of the market volume. Before proposing this approach to regulators as an efficient postmarket surveillance strategy, we recommend comparing results from different sampling approaches and conducting further analysis of the extent to which a sample, consisting of four different primary packaging and forty tablets or capsules, accurately represents a product.

A recent study in Indonesia reported that health workers, regulators and medicine producers share concerns that the pressure to reduce medicine prices in the public sector could lead to an increase in the exposure to poorly produced substandard medicines.^31^ Patients are also inclined to buy more expensive versions in the private market, assuming they are of better quality. We found no relationship between the price of amoxicillin and the results of assay and dissolution testing. This finding aligns with recent studies conducted in Indonesia and elsewhere, suggesting that significant savings could be achieved by procuring relatively cheaper product varieties.^66^,^68^

Our study also identified several problematic practices related to the provision of antibiotics that align with previous research in Indonesia and elsewhere.^13 25 69^ Mystery shoppers, also known as simulated patients, have frequently been used to purchase medicine samples in outlets,^43^ including unregulated channels.^25 35 63 70^ In the majority of outlets (94.6%), our mystery shoppers did not require a medical prescription when purchasing antibiotics. Additionally, amoxicillin was easily accessible in unlicensed outlets, including drug stores where selling antibiotics is prohibited. We also found that amoxicillin was readily available from numerous unlicensed online vendors, one of which provided the sample exhibiting the lowest dissolution profile.

Previous studies have mainly highlighted online purchases from unlicensed platforms with the associated risk of falsified and illegal medicines.^71^,^73^ Our study shows that this illicit practice also carries the risk of exposure to substandard products.^74 75^ Our results underscore the need for increased attention to counteract the unauthorised provision of antibiotics by unlicensed and online outlets. Addressing this issue requires collaboration among government agencies at both national and local levels. A key step is to prevent patients from resorting to unlicensed outlets by ensuring that medicines are adequately available in public facilities.^31 76^ Another promising strategy involves leveraging technology to increase transparency regarding medicines procurement, supply chain and availability.^77^

### Strengths and limitations

The strength of the study lies in the comprehensive survey of a substantial number of outlets (N=476) across four areas, including licensed, unlicensed and online vendors, and the diverse sample of amoxicillin oral solid products in the private market that represents 95% of the total market volume. The first limitation of this study is that although the 120 collected samples include 59 different products covering 95% of the estimated total market volume, numerous oral solid products authorised for the Indonesian market were still not included, including those in the public channels. Second, we only collected 10 dry syrup products in the Greater Jakarta area, which limits the understanding of quality assessment for the remaining products in the market.

### Future research

Future research could take into account diverse quality testing parameters for both active ingredients and excipients.^7 78^ Additionally, it is crucial to investigate the root cause of product degradation, differentiating it from substandard production.

## Conclusions

With the aim of collecting the largest possible variety of amoxicillin products available in the Indonesian market, we gathered 120 samples from licensed and unlicensed outlets in four areas, as well as from online vendors. 15 samples tested as OOS (12.5%). We found no relation between the price and quality of amoxicillin. Combining product-variety sampling with data on market volume presents a promising approach to gain insight into the prevalence of poor-quality medical products using a relatively small sample size.

## Supplementary material

10.1136/bmjopen-2024-093785 online supplemental file 1

10.1136/bmjopen-2024-093785 online supplemental file 2

10.1136/bmjopen-2024-093785 online supplemental file 3

10.1136/bmjopen-2024-093785 online supplemental file 4

10.1136/bmjopen-2024-093785 online supplemental file 5

10.1136/bmjopen-2024-093785 online supplemental file 6

10.1136/bmjopen-2024-093785 online supplemental file 7

10.1136/bmjopen-2024-093785 online supplemental file 8

## Data Availability

Data are available on reasonable request.
