# Supplementary material for: Assessing the quality of amoxicillin in the private market in Indonesia: a cross-sectional survey exploring product variety, market volume and price factors
Source: BMJ Open. 2025 Jul 22;15(7):e093785. doi: 10.1136/bmjopen-2024-093785 (PMC12306289; doi:10.1136/bmjopen-2024-093785)
Supplement: online supplemental file 1 [file bmjopen-15-7-s001.pdf]

Supplementary 1. MEDQUARG research checklist of items that should be addressed in reports of surveys of medicine quality

Section and topic	Item	Description	Reported on page number of not applicable (N/A)
Title/abstract/keywords	1	Identify the article as a study of medicine quality (Recommended MeSH headings 'medicine quality, substandard, degraded, counterfeit')	1
		Provide an abstract of what was done and what was found, describing the main survey methods and chemical analysis techniques used	2
Introduction	2	Summarise previous relevant drug quality information and describe the drug regulatory environment	4-5
		State specific objectives	5
**Methods**			
Survey details	3	The timing and location of the survey; when samples collected and when samples analysed	6, 9-10, Supplementary file 2
Definitions	4	The definitions of counterfeit, substandard and degraded medicines used	10
Outlets	5	The type, including indices of size (e.g. turnover), of drug outlets sampled	6-7
Sampling design	6	Sampling design and sample size calculation	7-8, not applicable for sample size calculation
		Type and number of dosage units purchased/outlet.	9
Samplers	7	Who carried out the sampling and in what guise.	9
		What did the collector say in buying the medicines?	9
		Definition of sampling frame.	7-8
		Question of interest, assumptions, sampling method(s) (including method of randomisation if random sampling used)	7-8, not applicable for randomisation
Statistical methods	8	Describe the data analysis techniques used.	11
Ethical issues	9	Whether ethical approval sought and whether the study encountered any ethical issues	11
Packaging	10	Packaging examination and reference standards	10, not applicable for packaging reference standards

Section and topic	Item	Description	Reported on page number of not applicable (N/A)
Chemical analysis	11	Chemical analysis and dissolution testing SOPs and location(s) of laboratory.<br><br>Description of validation and reference standards used	9-10, Supplementary file 4, Supplementary file 5<br><br>9-10, Supplementary file 4
Method validation	12	Details of laboratory method validation results, including but not limited to: Certificate of Analysis (COA) for reference standard, within and between run repeatability (RSD% for n=5-8), detection and quantitation limits, accuracy observed for reference samples, linear range for all analytes, sample preparation recovery studies, selectivity. Possibly, validation against a reference method or interlaboratory study.	Supplementary file 4, Supplementary file 5
Blinding	13	Whether chemistry was performed blinded to packaging and vice versa	10
**Results**			
Outlets	14	The details of the outlets actually sampled, 'class' of pharmacy (e.g., public, private for profit, private not or profit, informal, itinerant)	12
Missing samples	15	The reasons why any outlets chosen for sampling did not furnish a sample. Do these outlets differ systematically from those in which samples were obtained?	12
Packaging and chemistry results	16	Packaging and chemistry results and their relationship<br><br>Details of products sampled - how many, in what drug classes, countries of origin, batch numbers, manufacture and expiry dates<br><br>Results for each analysis – packaging, % AI, dissolution<br><br>Additional information could be included in Supplementary Material	13, not applicable for relationship<br><br>13-14<br><br>14-16
Category of poor-quality medicine	17	A clear statement for each medicine sample detected whether the investigators class it as genuine, counterfeit, substandard or degraded with an explanation as to why and whether the medicine was registered with the Government in the location(s) sampled	13

Section and topic	Item	Description	Reported on page number of not applicable (N/A)
State company and address as given on packaging	18	If the names of companies and addresses not given – to give a reason as to why this information is not provided.	13
Sharing data with MRA	19	Whether the data shared with the appropriate MRA and IMPACT	Yes, not in manuscript
Dissemination	20	Description of any non-covert packaging features that would allow others to detect counterfeit medicines. If publication is not possible, to consider disseminating via web-based Supplementary Material	Not applicable
**Discussion**			
Key results	21	Summarise key results with reference to study objectives	18
Limitations	22	Discussion of limitations of study, especially how robust the estimates of prevalence are and how applicable they may be to wider geographical areas.<br><br>Discuss the direction and extent of any potential bias	19-20<br><br>19-20
Interpretation	23	An interpretation of the results, in conjunction, with prior studies, in relation to public health	18-20
Intervention	24	Whether interventions are thought appropriate and, if so, what type	20
**Other Information**			
Conflict of interest	25	State any potential conflicts of interest	22
Funding	26	Give the source of funding and role of funders in the study	22
Supplementary information	27	If the journal allows, suggest to list important analytical methods and additional results which would allow others to replicate the work and compare with the reported study	Not applicable
