# Supplementary figures and images for: Assessing the quality of amoxicillin in the private market in Indonesia: a cross-sectional survey exploring product variety, market volume and price factors
Source: BMJ Open. 2025 Jul 22;15(7):e093785. doi: 10.1136/bmjopen-2024-093785 (PMC12306289; doi:10.1136/bmjopen-2024-093785)

## Supplementary 2. Sampling locations in Indonesia

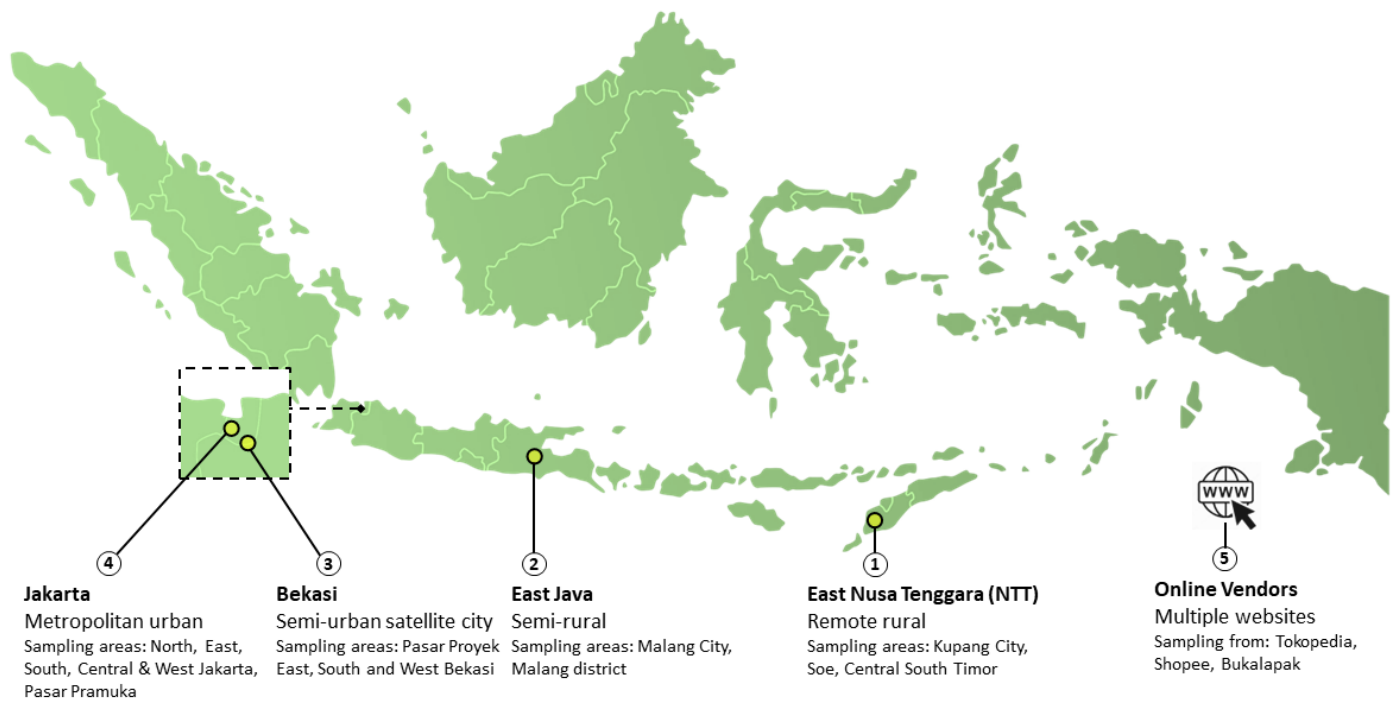

Supplement: online supplemental file 2 [file bmjopen-15-7-s002.pdf]
