# Supplementary material for: Assessing the quality of amoxicillin in the private market in Indonesia: a cross-sectional survey exploring product variety, market volume and price factors
Source: BMJ Open. 2025 Jul 22;15(7):e093785. doi: 10.1136/bmjopen-2024-093785 (PMC12306289; doi:10.1136/bmjopen-2024-093785)
Supplement: online supplemental file 3 [file bmjopen-15-7-s003.pdf]

# Supplementary 3. KoBo Collect forms

## Form A: To be completed by the data collector in the sampling locations or outlets

| Name | Label | Hint |
|---|---|---|
| start | | |
| end | | |
| today | | |
| deviceid | | |
| petugas | Data collector name | |
| petugas_lain | Data collector name | Data collector name if not on list - lowercase letters only |
| barcode | Scan barcode with your phone | |
| barcode_manual | Barcode manual | Manually enter barcode if scanning fails |
| provinsi | | |
| jakarta | Jakarta | |
| kota_jakarta | Areas in Jakarta where samples are collected | |
| kecamatan_jakut | Sub-district in North Jakarta where samples are collected | |
| kecamatan_jakbar | Sub-district in West Jakarta where samples are collected | |
| kecamatan_jaksel | Sub-district in South Jakarta where samples are collected | |
| kecamatan_jaktim | Sub-district in East Jakarta where samples are collected | |
| kecamatan_jakpus | Sub-district in Central Jakarta where samples are collected | |
| bekasi | Bekasi | |
| kecamatan_bekasi | Sub-district in Bekasi where samples are collected | |
| provinsi_lain | Non city | Fill internet if purchase on website |
| source_filter | Sampled in pharmacy or other? | |
| nama_apotek | Pharmacy name | |
| gis | GIS | Click "Start geopoint", then "Save geopoint" |
| source | Where did you get it? | |
| source_detail_lain | Name of other source | Fill the name where sample is purchased, if from internet fill the website's name |
| aircondition | Temperature | Are the medicines stored in an airconditioned room? |

| Name | Label | Hint |
| --- | --- | --- |
| **kesesuaian_sampel** | Is this sample the medicine you were targeting? | Think only of the sample covered by this barcode, not others bought at the same location |
| **Resep** | Prescription | Did the pharmacist ask for a prescription |
| **Resep_online** | Prescription online | Did the online vendor ask for a prescription? |
| **kasus_kontrol** | Is this sample a case or control? | Please check on the sampling form |
| **price** | Total price paid | If free, fill in 00 |
| **units** | Number of tablets obtained | How many tablets/bottles did you get for that price? |
| **notes_location** | Notes about the source location | Note anything unusual or relevant to meds quality (direct sunlight etc) |

## Form B: To be completed by the data collectors in the field office or base camp

| Name | Label | Hint |
|---|---|---|
| **start** | | |
| **end** | | |
| **today** | | |
| **deviceid** | | |
| **petugas** | Data collector name | |
| **petugas_lain** | Data collector name | Write if not on list - lowercase letters only |
| **barcode** | Scan the barcode with your phone | |
| **barcode_manual** | Manually enter barcode | Only necessary if scan does not work |
| **medicine** | Medicine sampled | |
| **amoxicillin** | Amoxicillin | You can buy Amoxicillin / Amoxicillin Trihydrate but NOT Co-Amoxicillin (with Clavulanate) |
| **amoxdose** | Dose | Asking about amoxicillin. Which dosage is each tablet? |
| **inn_amox** | Inn | Is it generic (with red/green stripes) or branded generic? |
| **merk_amox500_brand** | Merk | Brand name? |
| **merk_amox500_inn** | Permit holder | Name of Marketing Authorization (MA) holder -- company logo is usually on the packaging |
| **merk_amox250_brand** | Brand name | Brand name? |
| **merk_amox250_inn** | Permit holder | Name of Marketing Authorization (MA) holder -- company logo is usually on the packaging |
| **merk_amoxsirup125_brand** | Brand name | Brand name? |
| **merk_amoxsirup125_inn** | Company name | Name of Marketing Authorization (MA) holder -- company logo is usually on the packaging |
| **merk_amoxsirup250_brand** | Brand name | Brand name? |
| **merk_amoxsirup250_inn** | Company name | Name of Marketing Authorization (MA) holder -- company logo is usually on the packaging |
| **merk_amox_other_dose** | Other dose | Write in dose if not on list |
| **merk_amox_other_brand** | Brand name | Write in brand if not on list - lowercase letters only |

| Name | Label | Hint |
|---|---|---|
| **mf_amox_penyedia** | Permit holder | Write in Marketing Authorization (MA)  holder if generics - lowercase letters only |
| **mf_amox_produsen** | Producing factory | Write in producer name if different from permit holder (lowercase only) |
| **dossier_amox** | Dossier holder | Write in dossier holder if different from permit holder and there are more than 2 companies name (lowercase only) |
| **quantity_total** | Number of all tablets/bottles collected in a sample | |
| **pack** | Packaging | |
| **damage** | Condition | Is the packaging damaged? |
| **intact_pack** | Primary packaging condition | Is the primary packaging intact? For example, not deliberately cut. |
| **quantity_tab** | Number of tablets collected in one primary packaging | If the primary packaging not intact, please fill in with the most units. |
| **quantity_liq** | Number of ml collected in one primary packaging | |
| **nie** | NIE | Please fill without blank. Usually begins with DKL or GKL, followed by 13 numbers/letters. If illegible, please fill NA. |
| **batchnoflag** | Same batch number | Are all the medicines from the same batch? If not, mark for attention of supervisor |
| **batch** | Majority batch number | If more than one, enter number for batch with most pills |
| **batch_2** | Other batch number | |
| **expiry** | Expiry date | If none, please fill in 01/2015. If there's more than 1 batch number, fill in ED for first batch number. |
| **het** | Max retail price | Should be printed on the primary packaging. Enter numbers only. If missing, enter 00 |
| **foto_kemasaan_primer_strip_blister** | Photo of primary packaging | Brand name and permit holder should be legible |
| **foto_dus_atau_kemasaan_sekunder_lain** | Photo of secondary packaging | |
| **foto_nomor_bets** | Photo of batch number | If there's more than 1 batch number, fill in for first batch number. |
| **foto_tanggal_kadaluarsa** | Photo of expiry date | |
| **catatan_obat** | Notes on the sample | Note anything unusual about the sample here. For example, if the packaging was broken, give details |
| **online** | Do you buy this sample from internet? | |
| **onlinestore** | Name of website | Website's name where samples were purchased |
| **onlinestore_lain** | Name of other website | Website's name where samples were purchased if not on list - lowercase letters only |

| Name | Label | Hint |
|---|---|---|
| **vendor_account** | Name of vendor's account | Vendor's account name where samples were purchased |
| **orig_medicine** | Is this sample the medicine you were targeting? | Answer only for the samples with this barcode, not for other samples purchased at the same location |
| **original_medicine** | Original planned medicine | |
| **orig_amoxdose** | dose | Initial plan, sample which Amoxicillin dosage? |
| **orig_inn_amox** | inn | Is it generic or branded generic? |
