# Supplementary material for: Assessing the quality of amoxicillin in the private market in Indonesia: a cross-sectional survey exploring product variety, market volume and price factors
Source: BMJ Open. 2025 Jul 22;15(7):e093785. doi: 10.1136/bmjopen-2024-093785 (PMC12306289; doi:10.1136/bmjopen-2024-093785)
Supplement: online supplemental file 4 [file bmjopen-15-7-s004.pdf]

Equilab International

PHARMACEUTICAL ANALYSIS PROTOCOL

Pharmaceutical Analysis of Amoxicillin Tablet

Project No: AN 076/EQL/2020

Effective Date

Version No	Date
Version 1	11 January 2021

PT Equilab International
Jl. RS Fatmawati Persil 33
Jakarta 12430 – INDONESIA
Phone 62 21 7695513, 7515932
Fax 62 21 7509668

Signature Page

Approval by Equilab

Prepared by

11/01/2021

Deny Kurniawan
Analytical Method Development Officer

Date

Reviewed by

11/01/2021

Yantirta Indra Kurniawan, Chem
Bioanalytical Manager

Date

Approved by

11/01/2021

Ismail Dwi Saputro, Chem
Method Development and Validation Manager

Date

Verified and approved by

11/01/2021

Purnama Dewi Yuli Astuti, Pharm, B. Pharm
Quality Manager

Date

Approval by Sponsor

Approved by

15 June 2021

Amalia Hasnida, M.Sc
Researcher
Erasmus School of Health Policy & Management
Erasmus University Rotterdam

Date

Revision History

NO.	PART	CONTENT	REVISION DATE	VERSION NO.
1	-	New document	11/01/2021	1

1 ASSAY METHOD

Pharmaceutical analysis of amoxicillin tablet are performed in this present study for appearance, assay and dissolution.

The chemical structures of amoxicillin are shown in Figure 1.

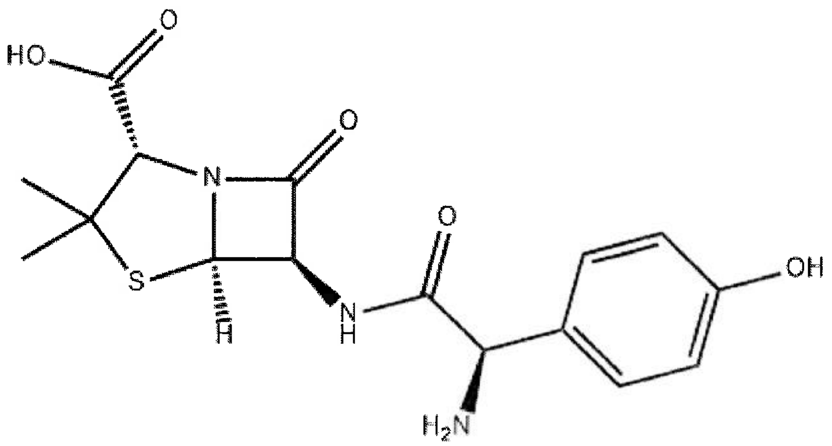

Figure 1. Chemical structures of amoxicillin

2 SAFETY

Laboratory coats must be worn all of the time during sample preparation, besides gloves should be worn when chemical solutions handling. Normal laboratory safety codes must be followed and the proper procedures have been adopted for the waste treatment.

3 MATERIAL

This protocol requires apparatus, standards and reagents as follows. The apparatus, standards or reagents which used in this study should be supplied by the proper manufacturers. Any changes in apparatus or reagents should be recorded as deviations from this protocol.

3.1 Apparatus

The required apparatus are listed in Table 1.

Table 1. Apparatus

**Apparatus**
Adjustable pipettes (10-100 µL, 100-1000 µL)
Disposable plastic pipette tips (range 10 – 100 µL and 100 – 1000 µL)
Volumetric flask
Beaker glass
Vortex mixer (Thermolyne)
Ultramicro Balance (Sartorius)
Analytical Balance (Mettler Toledo)
HPLC-UV (Waters, Aliance 2695 with UV Detector 2489)
Dissolution Tester (Hanson)
pH meter (VWR, pHenomenal)
Mechanical Shaker (D-LAB)
Ultrasonic (Elma S180H)
Filter RC 0.2 µm
Filter PVDF 0.45 µm
Centrifuge Universal (Hettich Zentrifugen 320)

3.2 Standard

The standards which be used in this validation method are listed in Table 2.

Table 2. Standard

Standard	Purpose	Source	Batch #	Purity	Storage	Expiry Date
Amoxicillin	Analyte	USP	R106H0	86.90%	Freezer (-20 ± 5 °C)	NA

3.3 Reagents

All of the used reagents are listed in Table 3.

Table 3. Reagents

Reagents	Source	Batch #	Storage	Expiry Date
Acetonitrile (ACN) gradient grade for LC	Merck	K52081430 006	Room temp (≤ 30 °C)	31/01/2023
Monobasic potassium phosphate (KH_2PO_4) for analysis	Merck	AM1358473 920	Room temp (≤ 30 °C)	31/10/2023
Potassium hydroxide (KOH) for analysis	Merck	B1148633 542	Room temp (≤ 30 °C)	NA
Deionized water	Arium UV Sartorius, In house	NA	Room temp (≤ 30 °C)	NA
Reverse Osmosis (RO) water	RO system, In house	NA	Room temp (≤ 30 °C)	NA

3.4 Product Samples

Table 4. Product Information

Product	Description	Note
Test	Amoxicillin 250 mg Tablet<br>Amoxicillin 500 mg Tablet	Product list was attached

4 PROCEDURES OF ANALYSIS

4.1 Appearance

Samples of tablets are collected at random and observe physically to check its appearance.

4.2 Assay of Amoxicillin

4.2.1 Reagent preparation

All the reagent that used in this process are prepared freshly or using solution that have been prepared before expired. The preparation procedures are described in details below.

4.2.1.1 Acetonitrile for mobile phase: pure acetonitrile freshly taken from its bottle.

4.2.1.2 Buffer solution: Weigh and dilute 6.8 g of KH_2PO_4 in 1000 mL of water. Adjust solution with 45% of KOH to a pH 5.0 ± 0.1 then mix well. Filter the solution using RC filter 0.2 µm then sonicate for 15 minutes.

4.2.1.3 Mobile phase = Acetonitrile:Buffer solution (1:24). Mix 20 mL of acetonitrile and 480 mL of buffer solution. Stirer the solution for 15 minutes and then sonicate for 15 minutes. The volume of each solvent can be changed but the ratio should be same.

4.2.2 Standard Solution of USP Amoxicillin RS 1.2 mg/mL

4.2.2.1 Weigh 12.0 mg of USP Amoxicillin RS on aluminium foil then put it into 10 mL volumetric flask.

4.2.2.2 Dilute with buffer solution to volume and mix well.

4.2.3 Sample Solution (Concentration of Amoxicillin anhydrous 1 mg/mL)

4.2.3.1 Place not less than 5 tablets into volumetric flask. Add buffer into ¾ volume.

4.2.3.2 Sonicate for 5 minutes and dilute with buffer to volume and mix well with magnetic stirring bar and stir for 30 minutes.

4.2.3.3 Centrifuge a portion of solution 4000 rpm for 5 minutes.

4.2.3.4 Filter with PVDF 0.45 µm. Discard the first few mL of the filtrate.

4.2.3.5 Use this solution within 6 hours.

4.2.4 Chromatographic Condition

4.2.4.1 Mode : LC
4.2.4.2 Detector : UV 230 nm
4.2.4.3 Column : Merck, LiChrospher® 100, C18, 4.0 x 250 mm, 5 µm
4.2.4.4 Mobile phase : Acetonitrile:Buffer (1:24)
4.2.4.5 Flow rate : 1.5 mL/min
4.2.4.6 Injection Volume : 10 µL

4.2.5 System suitability

4.2.5.1 Tailing factor NMT 2.5.
4.2.5.2 Relative standard deviation (RSD) NMT 2.0%.

4.2.6 Procedure

4.2.6.1 Prepare the sample solution in duplicate.
4.2.6.2 Record the chromatogram for all injections as per below sequence

No	Name of Solution	No. of injection
1	Blank (Diluent/Mobile phase)	1
2	Standard solution	5
3	Sample solution 1	1
4	Sample solution 2	1
5	Standard solution (Bracketing)	2

4.2.7 Calculation

The percentage assay of Amoxicillin in Amoxicillin Tablet is calculated by the following formula

$= (A_{spl}/A_{std}) \times (C_{std}/C_{spl}) \times P \times F \times 100$

A_{spl} : peak area of sample solution
A_{std} : peak area of standard solution
C_{std} : Concentration of USP Amoxicillin RS in standard solution (mg/mL)
C_{spl} : Nominal concentration of Amoxicillin in sample solution (mg/mL)
P : Potency of amoxicillin in USP Amoxicillin RS (µg/mg)
F : Conversion factor, 0.001 mg/ µg

4.3 Dissolution of Amoxicillin

4.3.1 Reagent preparation

All the reagent that used in this process are prepared freshly or using solution that have been prepared before expired. The preparation procedures are described in details below.

4.3.1.1 Acetonitrile for mobile phase: pure acetonitrile freshly taken from its bottle.

4.3.1.2 Buffer solution: Weigh 27.2 g of KH_2PO_4 in 3000 mL of water. Adjust solution with 45% KOH to a pH 5.0 ± 0.1. Dilute with water into 4000 mL and mix well. Filter the solution using RC filter 0.2 µm then sonicate for 15 minutes.

4.3.1.3 Mobile phase = Acetonitrile:Buffer solution (1:39). Mix 20 mL of acetonitrile and 780 mL buffer solution. Stirer the solution for 15 minutes and then sonicate for 15 minutes. The volume of each solvent can be changed but the ratio should be same.

4.3.2 Standard solution of USP Amoxicillin RS 0.05 mg/mL

4.3.2.1 Weigh 1 mg of USP Amoxicillin RS on aluminium foil then put it into 20 mL volumetric flask.

4.3.2.2 Dilute with buffer solution to volume and mix well.

4.3.3 Sample Dissolution

4.3.3.1 Set dissolution tester equipment at temperature 37 ± 0.5 °C.

4.3.3.2 Transfer 900 mL of dissolution media into No 1 - 6 vessels.

4.3.3.3 Weighing 6 tablets of sample with analytical balance.

4.3.3.4 After the temperature reached 37 ± 0.5 °C on each vessel located the tablets above dissolution cover.

4.3.3.5 Enter sample into vessel.

4.3.3.6 Push '*run*' at dissolution tester.

4.3.3.7 Take 10 mL of solution in dissolution vessels as sampling time.

4.3.3.8 Filter with PVDF 0.45 µm. Discard the first few mL of the filtrate.

4.3.3.9 Dilute a volume of filtrate with water to obtained an estimated concentration of 0.045 mg/mL of amoxicillin (pipette 2 mL into 5 mL volumetric flask for Amoxicillin 125 mg tablet, pipette 1 mL into 5 mL volumetric flask for Amoxicillin 250 mg tablet, pipette 0.5 mL into 5 mL volumetric flask for Amoxicillin 500 mg tablet).

4.3.3.10 Use this solution within 6 hours

4.3.4 Chromatographic Condition

4.3.4.1 Mode : LC
4.3.4.2 Detector : UV 230 nm
4.3.4.3 Column : Merck, LiChrospher® 100, C18, 4.0 x 250 mm, 5 µm
4.3.4.4 Column temp. : 40 ± 1 °C
4.3.4.5 Mobile phase : Acetonitrile:Buffer (1:39)
4.3.4.6 Flow rate : 0.6 mL/min
4.3.4.7 Injection Volume : 10 µL

4.3.5 Dissolution tester condition

4.3.5.1 Apparatus : USP 2 Paddle, 75 rpm
4.3.5.2 Dissolution medium : Water, 900 mL
4.3.5.3 Sampling time : 30 minutes
4.3.5.4 Sampling volume : 10 mL
4.3.5.5 Temperature : 37 ± 0.5 °C

4.3.6 System suitability

4.3.6.1 Tailing factor NMT 2.5.
4.3.6.2 Relative standard deviation (RSD) NMT 1.5%.

4.3.7 Procedure

4.3.7.1 Record the chromatogram for all injections as per below sequence

No	Name of Solution	No. of Injection
1	Standard solution	5
2	Sample dissolution 1	1
3	Sample dissolution 2	1
4	Sample dissolution 3	1
5	Sample dissolution 4	1
6	Sample dissolution 5	1
7	Sample dissolution 6	1
8	Standard solution (Bracketing)	2

4.3.8 Calculation

The percentage assay of Amoxicillin in Amoxicillin Tablet is calculated by the following formula

$= (A_{spl}/A_{std}) \times (C_{std}/L) \times V \times D \times P \times F \times 100$

A_{spl}	: peak area of sample solution
A_{std}	: peak area of standard solution
C_{std}	: Concentration of USP Amoxicillin RS in standard solution (mg/mL)
C_{spl}	: Nominal concentration of Amoxicillin in sample solution (mg/mL)
L	: Label claim (mg/Tablet)
V	: Volume of the dissolution medium, 900 mL
D	: Dilution factor for the sample solution
P	: Potency of amoxicillin in USP Amoxicillin RS (µg/mg)
F	: Conversion factor, 0.001 mg/ µg

5 SPECIFICATION

Table 5. Product Specification

No	Test	Specification
1	Appearance	NA
2	Assay	90.0% - 120.0%
3	Dissolution	NLT 75% (Q) of the labeled amount of amoxicillin ($C_{16}H_{19}N_3O_5S$) is dissolved

6 PROTOCOL DEVIATION

All protocol deviation will be recorded and reported in the analytical report

7 REFERENCES

US Pharmacopeia. USP 42, NF 37, Amoxicillin Tablets, page 299

Equilab International

PHARMACEUTICAL ANALYSIS PROTOCOL

Pharmaceutical Analysis of Amoxicillin Capsules

Project No: AN 076/EQL/2020

Effective Date

Version No	Date
Version 1	25 March 2021

PT Equilab International
Jl. RS Fatmawati Persil 33
Jakarta 12430 – INDONESIA
Phone 62 21 7695513, 7515932
Fax 62 21 7509668

Signature Page

Approval by Equilab

Prepared by

Deny Kurniawan
Analytical Method Development Officer

25/03/2021
Date

Reviewed by

Yantirta Indra Kurniawan, Chem
Bioanalytical Manager

25/03/2021
Date

Approved by

Ismail Dwi Saputro, Chem
Method Development and Validation Manager

25/03/2021
Date

Verified and approved by

Purnama Dewi Yuli Astuti, Pharm, B. Pharm
Quality Manager

25/03/2021
Date

Approval by Sponsor

Approved by

Amalia Hasnida, M.Sc
Researcher
Erasmus School of Health Policy & Management
Erasmus University Rotterdam

10 June 2021
Date

Revision History

NO.	PART	CONTENT	REVISION DATE	VERSION NO.
1	-	New document	25/03/2021	1

1 ANALYSIS

Pharmaceutical analysis of amoxicillin capsules is performed in this present study for appearance, identification, assay and dissolution.

The chemical structures of amoxicillin are shown in Figure 1.

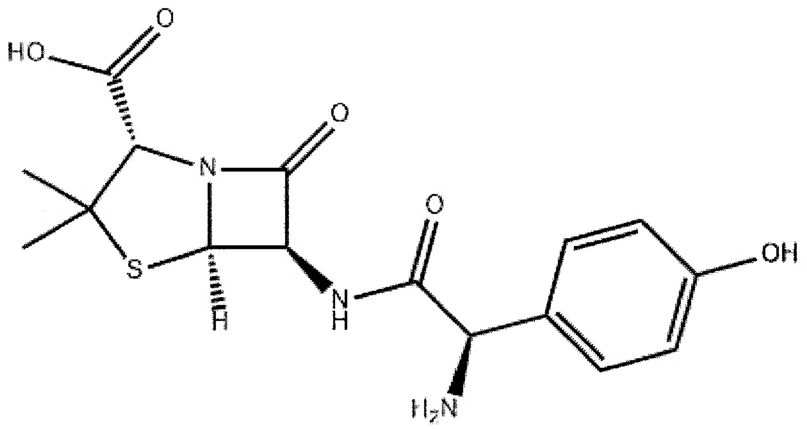

Figure 1. Chemical structures of amoxicillin

2 SAFETY

Laboratory coats must be worn all of the time during sample preparation, besides gloves should be worn when chemical solutions handling. Normal laboratory safety codes must be followed, and the proper procedures have been adopted for the waste treatment.

3 MATERIAL

This protocol requires apparatus, standards and reagents as follows. The apparatus, standards or reagents which used in this study should be supplied by the proper manufacturers. Any changes in apparatus or reagents should be recorded as deviations from this protocol.

3.1 Apparatus

The required apparatus is listed in Table 1.

Table 1. Apparatus

Apparatus
Adjustable pipettes (10-100 µL, 100-1000 µL)
Disposable plastic pipette tips (range 10 – 100 µL and 100 – 1000 µL)
Volumetric flask
Vortex mixer (Thermolyne)
Ultramicro Balance (Sartorius)
Analytical Balance (Mettler Toledo)
HPLC-UV (Waters, Alliance 2695 with UV Detector 2489)
Spectrophotometer-UV/VIS (Shimadzu UV-1800)
Dissolution Tester (Hanson)
pH meter (VWR, pHenomenal)
Ultrasonic (Elma S180H)
Filter RC 0.2 µm
Filter PVDF 0.45 µm
Centrifuge Universal (Hettich Zentrifugen 320)

3.2 Standard

The standards which be used in this pharmaceutical analysis are listed in Table 2.

Table 2. Standard

Standard	Purpose	Source	Batch #	Purity	Storage	Expiry Date
Amoxicillin	Analyte	USP	R106H0	86.90%	Freezer (-20 ± 5°C)	NA

3.3 Reagents

All of the used reagents are listed in Table 3.

Table 3. Reagents

Reagents	Source	Batch #	Storage	Expiry Date
Acetonitrile (ACN) gradient grade for LC	Merck	K52081430 006	Room temp (≤ 30°C)	31/01/2023
Monobasic potassium phosphate (KH_2PO_4) for analysis	Merck	AM1358473 920	Room temp (≤ 30°C)	31/10/2023
Potassium hydroxide (KOH) for analysis	Merck	B1890433 108	Room temp (≤ 30°C)	31/10/2023
Deionized water	Arium UV Sartorius, In house	NA	Room temp (≤ 30°C)	NA
Reverse Osmosis (RO) water	RO system, In house	NA	Room temp (≤ 30°C)	NA

3.4 Product Samples

Table 4. Product Information

Product	Description	Note
Test	Amoxicillin 250 mg Capsules<br>Amoxicillin 500 mg Capsules	Product list was attached

4 PROCEDURES OF ANALYSIS

4.1 Appearance

Samples of capsules are collected at random and observe physically to check its appearance.

4.2 Identification

The retention time of the major of the sample solution corresponds to that of the standard solution as obtained in the assay.

4.3 Assay of Amoxicillin

4.3.1 Reagent preparation

All the reagent that used in this process are prepared freshly or using solution that have been prepared before expired. The preparation procedures are described in detail below.

4.3.1.1 Acetonitrile for mobile phase: pure acetonitrile freshly taken from its bottle.

4.3.1.2 Buffer solution: Weigh and dilute 6.8 g of KH_2PO_4 in 1000 mL of water. Adjust solution with 45% of KOH to a pH 5.0 ± 0.1 then mix well. Filter the solution using RC filter 0.2 µm then sonicate for 15 minutes.

4.3.1.3 Mobile phase = Acetonitrile: Buffer solution (1:24). Mix 20 mL of acetonitrile and 480 mL of buffer solution. Stirrer the solution for 15 minutes and then sonicate for 15 minutes. The volume of each solvent can be changed but the ratio should be same.

4.3.2 Standard Solution of USP Amoxicillin RS 1.2 mg/mL

4.3.2.1 Investigate the purity of reference standard from CoA to determine the weighing of standard.

4.3.2.2 Weigh 12.0 mg of USP Amoxicillin RS on aluminum foil then put it into 10 mL volumetric flask.

4.3.2.3 Dilute with buffer solution to volume and mix well.

4.3.2.4 Use this solution within 6 hours.

4.3.3 Sample Solution (Concentration of Amoxicillin anhydrous 1 mg/mL)

4.3.3.1 Remove, as completely as possible, the content of not less than 20 capsules

4.3.3.2 Mix the combined contents

4.3.3.3 Transfer a quantity equivalent to 200 mg of anhydrous amoxicillin to a 200 mL volumetric flask. Add buffer to volume

4.3.3.4 Sonicate for around 5 minutes to ensure complete dissolution

4.3.3.5 Use this solution within 6 hours.

4.3.4 Chromatographic Condition

4.3.4.1 Mode	: LC
4.3.4.2 Detector	: UV 230 nm
4.3.4.3 Column	: Waters, Sunfire® C18,<br>4 x 250 mm, 10 µm

4.3.4.4 Mobile phase : Acetonitrile: Buffer (1:24)
4.3.4.5 Flow rate : 1.5 mL/min
4.3.4.6 Injection Volume: 10 µL

4.3.5 System suitability

4.3.5.1 Tailing factor NMT 2.5.
4.3.5.2 Relative standard deviation (RSD) NMT 2.0%.

4.3.6 Procedure

4.3.6.1 Prepare the sample solution in duplicate.
4.3.6.2 Record the chromatogram for all injections as per below sequence

No	Name of Solution	No. of injection
1	Blank (Diluent/Mobile phase)	1
2	Standard solution	5
3	Sample solution A 1	1
4	Sample solution A 2	1
5	Standard solution (Bracketing)	2
6	Sample solution n 1	1
7	Sample solution n 2	1
8	Standard solution (Bracketing)	2
9	etc (if any)	-

4.3.7 Calculation

The percentage assay of labeled amount of amoxicillin ($C_{16}H_{19}N_3O_5S$) in the portion of capsules taken is calculated by the following formula:

$$= (A_{spl}/A_{std}) \times (C_{std}/C_{spl}) \times P \times F \times 100$$

A_{spl} : peak area of sample solution
A_{std} : peak area of standard solution
C_{std}: Concentration of USP Amoxicillin RS in standard solution (mg/mL)
C_{spl} : Nominal concentration of Amoxicillin in sample solution (mg/mL)
P : Potency of amoxicillin in USP Amoxicillin RS (µg/mg)
F : Conversion factor, 0.001 mg/ µg

CONFIDENTIAL

4.4 Dissolution of Amoxicillin

4.4.1 Reagent preparation

All the reagent that used in this process are prepared freshly or using solution that have been prepared before expired. The preparation procedures are described in detail below.

4.4.1.1 Water: use the reverse osmosis (RO) water for dissolution medium

4.4.2 Standard solution of USP Amoxicillin RS 0.05 mg/mL

4.4.2.1 Investigate the purity of reference standard from CoA to determine the weighing of standard.

4.4.2.2 Weigh 1 mg of USP Amoxicillin RS on aluminum foil then put it into 20 mL volumetric flask.

4.4.2.3 Dilute with dissolution medium to volume and mix well.

4.4.3 Sample Dissolution

4.4.3.1 Set dissolution tester equipment at temperature 37 ± 0.5°C.

4.4.3.2 Transfer 900 mL of dissolution media into No 1 - 6 vessels.

4.4.3.3 Weighing 6 capsules of sample with analytical balance.

4.4.3.4 After the temperature reached 37 ± 0.5°C on each vessel located the Capsules above dissolution cover.

4.4.3.5 Enter sample into vessel.

4.4.3.6 Push '*run*' at dissolution tester.

4.4.3.7 Take 10 mL of solution in dissolution vessels as sampling time.

4.4.3.8 Filter with PVDF 0.45 µm. Discard the first few mL of the filtrate.

4.4.3.9 Dilute a volume of filtrate with water to obtain an estimated concentration of 0.05 mg/mL of amoxicillin (pipette 900 µL into 5 mL volumetric flask for Amoxicillin 250 mg capsules, pipette 450 µL into 5 mL volumetric flask for Amoxicillin 500 mg capsules).

4.4.4 Spectrophotometer Condition

4.4.4.1 Detector : UV-Vis

4.4.4.2 Wavelength : 272 nm

4.4.5 Dissolution tester condition

4.4.5.1 Apparatus : USP 1 Basket, 100 rpm (for 250 mg capsules)

		USP 2 Paddle, 75 rpm (for 500 mg capsules)
4.4.5.2	Dissolution medium	: Water, 900 mL
4.4.5.3	Sampling time	: 60 minutes
4.4.5.4	Sampling volume	: 10 mL
4.4.5.5	Temperature	: 37 ± 0.5 °C

4.4.6 System suitability

-

4.4.7 Procedure

4.4.7.1 Take absorbance for all samples as per below sequence

No	Name of Solution	No. of Absorbance
1	Standard solution	5
2	Sample dissolution 1	1
3	Sample dissolution 2	1
4	Sample dissolution 3	1
5	Sample dissolution 4	1
6	Sample dissolution 5	1
7	Sample dissolution 6	1
8	Standard solution (Bracketing)	2
9	Sample dissolution n 1	1
10	Sample dissolution n 2	1
11	Sample dissolution n 3	1
12	Sample dissolution n 4	1
13	Sample dissolution n 5	1
14	Sample dissolution n 6	1
15	Standard solution (Bracketing)	2
16	etc (if any)	-

4.4.8 Calculation

The percentage of label amount of Amoxicillin ($C_{16}H_{19}N_3O_5S$) dissolved is calculated by the following formula

$$= (A_{spl}/A_{std}) \times (C_{std}/L) \times V \times D \times P \times F \times 100$$

A_{spl} : peak area of sample solution

A_{std} : peak area of standard solution

C_{std} : Concentration of USP Amoxicillin RS in standard solution (mg/mL)

C_{spl} : Nominal concentration of Amoxicillin in sample solution (mg/mL)

L : Label claim (mg/Capsule)

V : Volume of the dissolution medium, 900 mL

D : Dilution factor for the sample solution

P : Potency of amoxicillin in USP Amoxicillin RS (µg/mg)

F : Conversion factor, 0.001 mg/ µg

5 SPECIFICATION

Table 5. Product Specification

No	Test	Specification
1	Appearance	NA
2	Identification	RT of sample peak as standard assay
3	Assay	90.0% - 120.0% of the labeled amount of amoxicillin ($C_{16}H_{19}N_3O_5S$)
4	Dissolution	NLT 80% (Q) of the labeled amount of amoxicillin ($C_{16}H_{19}N_3O_5S$) is dissolved

6 PROTOCOL DEVIATION

All protocol deviation will be recorded and reported in the analytical report

7 REFERENCES

US Pharmacopeia. USP 42, NF 37, Amoxicillin Capsules, page 295

Equilab International

PHARMACEUTICAL ANALYSIS PROTOCOL

Pharmaceutical Analysis of Amoxicillin for Oral Suspension (DS/Dry Syrup)

Project No: AN 076/EQL/2020

Effective Date

**Version No**	**Date**
Version 1	08 Nov 2021

PT Equilab International
Jl. RS Fatmawati Persil 33
Jakarta 12430 – INDONESIA
Phone: 62 21 7695513, 7515932
Fax: 62 21 7509668

Signature Page

Approval by Equilab

Prepared by

	08/11/2021
Mellisa Martina, Eng<br>Analytical Method Development Officer	Date

Reviewed by

	08/11/2021
Yantirta Indra Kurniawan, Chem<br>Bioanalytical Manager	Date

Approved by

	08/11/2021
Ismail Dwi Saputro, Chem<br>Method Development and Validation Manager	Date

Verified and approved by

	08/11/2021
Purnama Dewi Yuli Astuti, Pharm, B. Pharm<br>Quality Manager	Date

Approval by Sponsor

Approved by

	8 November 2021
Amalia Hasnida, M.Sc.,<br>Researcher<br>Erasmus School of Health Policy & Management<br>Erasmus University Rotterdam	Date

Revision History

NO.	PART	CONTENT	REVISION DATE	VERSION NO.
1	-	New document	08/11/2021	1

ANALYSIS

Pharmaceutical analysis of amoxicillin DS is performed in this present study for appearance, identification, and assay.

The chemical structures of amoxicillin are shown in Figure 1.

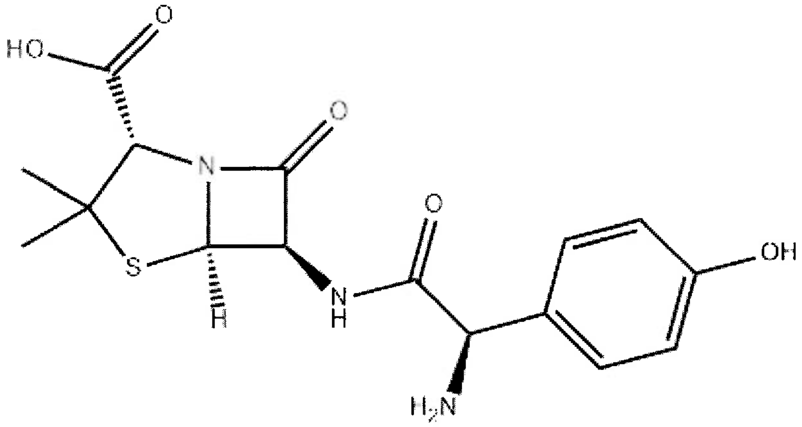

Figure 1. Chemical structures of amoxicillin

SAFETY

Laboratory coats must be worn all of the time during sample preparation, besides gloves should be worn when chemical solutions handling. Normal laboratory safety codes must be followed, and the proper procedures have been adopted for the waste treatment.

MATERIAL

This protocol requires apparatus, standards and reagents as follows. The apparatus, standards or reagents which used in this study should be supplied by the proper manufacturers. Any changes in apparatus or reagents should be recorded as deviations from this protocol.

3.1 Apparatus

The required apparatus is listed in Table 1.

Table 1. Apparatus

Apparatus
Adjustable pipettes (10-100 μL, 100-1000 μL)
Disposable plastic pipette tips (range 10 – 100 μL and 100 – 1000 μL)
Volumetric flask
Vortex mixer (Thermolyne)
Ultramicro Balance (Sartorius)
Analytical Balance (Mettler Toledo)
HPLC-UV (Waters, Alliance 2695 with UV Detector 2489)
pH meter (VWR, pHenomenal)
Ultrasonic (Elma S180H)
Filter RC 0.2 μm
Filter PVDF 0.45 μm

3.2 Standard

The standards which be used in this pharmaceutical analysis are listed in Table 2.

Table 2. Standard

Standard	Purpose	Source	Batch #	Purity	Storage	Expiry Date
Amoxicillin	Analyte	USP	R106H0	86.90%	Freezer (-20 ± 5°C)	NA

3.3 Reagents

All of the used reagents are listed in Table 3.

Table 3. Reagents

Reagents	Source	Batch #	Storage	Expiry Date
Acetonitrile (ACN) gradient grade for LC	Merck	K52081430 006	Room temp (≤ 30°C)	31/01/2023
Monobasic potassium phosphate (KH_2-PO_4) for analysis	Merck	AM1358473 920	Room temp (≤ 30°C)	31/10/2023
Potassium hydroxide (KOH) for analysis	Merck	B1890433 108	Room temp (≤ 30°C)	31/10/2023
Deionized water	Arium UV Sartorius, In house	NA	Room temp (≤ 30°C)	NA

3.4 Product Samples

Table 4. Product Information

Product	Description	Note
Test	Amoxicillin 125 mg/5 mL (60 mL)<br>Amoxicillin 250 mg/5 mL (60 mL)	Product list was attached

PROCEDURES OF ANALYSIS

4.1 Appearance

Samples of DS are collected at random and observe physically to check its appearance.

4.2 Identification

The retention time of the major peak of the sample solution corresponds to that of the standard solution as obtained in the assay.

4.3 Assay of Amoxicillin

4.3.1 Reagent preparation

All the reagent that used in this process are prepared freshly or using solution that have been prepared before expired. The preparation procedures are described in detail below.

4.3.1.1 Acetonitrile for mobile phase: pure acetonitrile freshly taken from its bottle.

4.3.1.2 Buffer solution: Weigh and dilute 6.8 g of KH_2PO_4 in 1000 mL of water. Adjust solution with 45% (w/w) solution of KOH to a pH 5.0 ± 0.1 then mix well. Filter the solution using RC filter 0.2 µm then sonicate for 15 minutes.

4.3.1.3 Mobile phase = Acetonitrile: Buffer solution (1:24). Mix 20 mL of acetonitrile and 480 mL of buffer solution. Stirrer the solution for 15 minutes and then sonicate for 15 minutes. The volume of each solvent can be changed but the ratio should be same.

4.3.2 Standard Solution of USP Amoxicillin RS 1.2 mg/mL

4.3.2.1 Investigate the purity of reference standard from CoA to determine the weighing of standard.

4.3.2.2 Weigh 12.0 mg of USP Amoxicillin RS on aluminum foil then put it into 10 mL volumetric flask.

4.3.2.3 Dilute with buffer solution to volume and mix well.

4.3.2.4 Use this solution within 6 hours

4.3.3 Sample Solution (Concentration of Amoxicillin anhydrous 1 mg/mL)

4.3.3.1 Dilute a measured volume of Amoxicillin DS, constituted as directed in the labeling.

4.3.3.2 Transferred all sample into volumetric flask (250 mL volumetric flask for amoxicillin 125 mg DS and 500 mL volumetric flask for amoxicillin 250 mg DS).

4.3.3.3 Rinse the bottle sample with 5 x 10 mL buffer solution (note: make sure all sample has been transferred into volumetric flask).

4.3.3.4 Add buffer solution into ¾ volume of volumetric flask.

4.3.3.5 Sonicate for 10 minutes with intermittent shaking per 2 minutes. Allow to room temperature and add buffer solution into volume.

4.3.3.6 Pipette 1667 µL of solution into 10 mL volumetric flask. Add buffer solution into volume and mix homogeneously.

4.3.3.7 Filter with PVDF 0.45 µm. Discard the first few mL of the filtrate.

4.3.3.8 Use this solution within 6 h.

4.3.4 Chromatographic Condition

4.3.4.1 Mode : LC

4.3.4.2 Detector : UV 230 nm
4.3.4.3 Column : Waters, Sunfire® C18, 4.0 x 250 mm, 10 μm
4.3.4.4 Mobile phase : Acetonitrile: Buffer solution (1:24)
4.3.4.5 Flow rate : 1.5 mL/min
4.3.4.6 Injection Volume : 10 μL

4.3.5 System suitability

4.3.5.1 Use the standard solution.
4.3.5.2 Tailing factor NMT 2.5.
4.3.5.3 Relative standard deviation (RSD) NMT 2.0%.

4.3.6 Procedure

4.3.6.1 Prepare the sample solution in duplicate.
4.3.6.2 Record the chromatogram for all injections as per below sequence

No	Name of Solution	No. of injection
1	Blank (Diluent/Mobile phase)	1
2	Standard solution	5
3	Sample solution A 1	1
4	Sample solution A 2	1
5	Standard solution (Bracketing)	2
6	Sample solution n 1	1
7	Sample solution n 2	1
8	Standard solution (Bracketing)	2
9	etc (if any)	-

4.3.7 Calculation

The percentage of amoxicillin ($C_{16}H_{19}N_3O_5S$) in the Amoxicillin DS is calculated by the following formula:

$= (A_{spl}/A_{std}) \times (C_{std}/C_{spl}) \times P \times F \times 100$

A_{spl} : peak area of sample solution
A_{std} : peak area of standard solution
C_{std} : Concentration of USP Amoxicillin RS in standard solution (mg/mL)
C_{spl} : Nominal concentration of anhydrous amoxicillin in sample solution (mg/mL)
P : Potency of amoxicillin in USP Amoxicillin RS (μg/mg)

CONFIDENTIAL

F : Conversion factor, 0.001 mg/µg

SPECIFICATION

Table 5. Product Specification

No	Test	Specification
1	Appearance	NA
2	Identification	RT of sample peak as standard assay
3	Assay	90.0% - 120.0% of the labeled amount of amoxicillin ($C_{16}H_{19}N_3O_5S$)

PROTOCOL DEVIATION

All protocol deviation will be recorded and reported in the analytical report

REFERENCES

US Pharmacopeia. USP 42, NF 37, Amoxicillin for Oral Suspension, page 298
