# Supplementary material for: Assessing the quality of amoxicillin in the private market in Indonesia: a cross-sectional survey exploring product variety, market volume and price factors
Source: BMJ Open. 2025 Jul 22;15(7):e093785. doi: 10.1136/bmjopen-2024-093785 (PMC12306289; doi:10.1136/bmjopen-2024-093785)
Supplement: online supplemental file 5 [file bmjopen-15-7-s005.pdf]

Supplementary 5. Quality testing parameter and limits of compliance of amoxicillin by dosage forms (based on United States Pharmacopeia 42 National Formulary 37)

Dosage forms	Quality testing parameter					
	Identification	Assay	Dissolution		Numbers of units tested	
			[Q]%	[Q + 5] %	Dissolution Stage 1 (S_1)	Dissolution Stage 2 (S_2)
**Tablet**	RT* of sample peak as standard assay %	90-120	75**	80	6	12
**Capsule**	RT of sample peak as standard assay %	90-120	80***	85	6	12
**Dry syrup**	RT of sample peak as standard assay %	90-120	Not applicable	Not applicable	Not applicable	Not applicable

*RT = sum of all the peak responses from the Sample solution
**For tablets, 75% of the labelled active ingredients should be dissolved within 30 minutes
***For capsules, 80% of the labelled active ingredients should be dissolved within 60 minutes
