# Supplementary material for: Assessing the quality of amoxicillin in the private market in Indonesia: a cross-sectional survey exploring product variety, market volume and price factors
Source: BMJ Open. 2025 Jul 22;15(7):e093785. doi: 10.1136/bmjopen-2024-093785 (PMC12306289; doi:10.1136/bmjopen-2024-093785)
Supplement: online supplemental file 6 [file bmjopen-15-7-s006.pdf]

Supplementary 6.

Amoxicillin oral solid (N=95) and dry syrup (N=79) product variety and estimated volume market share in the Indonesian market in the year proceeding sampling (October 2019-September 2020)

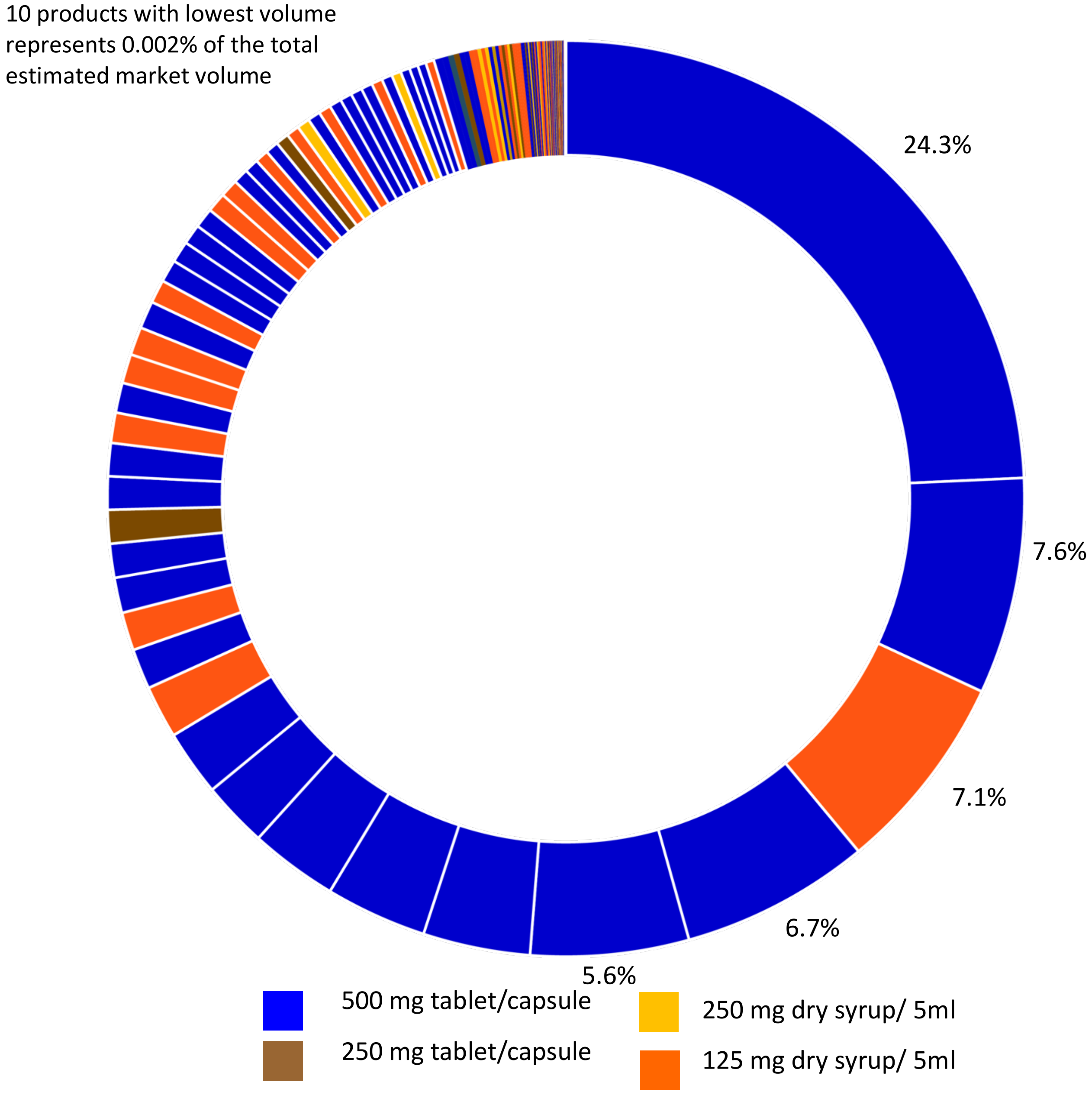

Source: This is based on internal analysis by (Hasnida et al., 2025) using data from the following source: IQVIA MIDAS Quarterly Sales for the period (October 2019-September 2020) reflecting estimates of real-world activity. Copyright IQVIA. All rights reserved.
