# Supplementary material for: Assessing the quality of amoxicillin in the private market in Indonesia: a cross-sectional survey exploring product variety, market volume and price factors
Source: BMJ Open. 2025 Jul 22;15(7):e093785. doi: 10.1136/bmjopen-2024-093785 (PMC12306289; doi:10.1136/bmjopen-2024-093785)
Supplement: online supplemental file 7 [file bmjopen-15-7-s007.pdf]

Supplementary file 7. Outlets visited, product varieties identified, and collected samples of oral solid by area (source: field work data & pharmaceutical market data)

	Areas					
	**1: NTT**	**2: East Java**	**3: Jakarta**	**4: Bekasi**	**5: Online**	**Total**
	**Outlets for product survey**					
**Outlets visited**	47	132	144	59	94**	**476**
Pharmacy	46	126	101	53		326
Drug store (not allowed to sell amoxicillin)*	1	2	43	6		52
Health provider * (not allowed to sell amoxicillin)	0	4	0	0		4
	**Distinct product[1] variety**					
**Total product variety in visited outlets**	15	38	32	31	18***	
Unique products not identified in previous area	15	24	7	2	9	
Estimated market volume of identified product variety	220 M	332 M	291 M	297 M	546 M	
	**Samples[2] collected & product variety**					
**Total samples & products collected in visited outlets**						
Number of samples collected	14	34	29	18	15	**110**
Total product variety of collected samples	7	27	24	15	13	
Unique products[3] collected	7	21	10	4	7	
Estimated market volume of collected products	44 M	281 M	235 M	71 M	51 M	
	**Outlets for samples collection**					
**Total outlets from which samples were collected**	11	32	22	16	15	**96**
Pharmacy	10	26	9	14		59
Drug store	1	2	13	2		18
Health provider (i.e., physicians & midwives)	0	4	0	0		4
**Samples obtained without prescriptions**	11 (78.6%)	32 (94.1%)	28 (96.6%)	18 (100%)	13 (86.7%)	**112 (93.3%)**

1 Distinct product: one amoxicillin brand, of one dosage, in tablets or capsules, and from one manufacturer

2 Sample: one amoxicillin product, collected at one location, at one time

3 Unique products: newly collected product varieties previously not identified in the previous areas

*Not allowed to sell prescription-only medicines

*** Number of interactions with the vendors

*** Product identifications were limited to search for remaining unidentified products in the previous areas

Source: This is based on internal analysis by (Hasnida et al., 2025) using data from the following source: IQVIA MIDAS Quarterly Sales for the period (October 2019-September 2020) reflecting estimates of real-world activity. Copyright IQVIA. All rights reserved.
