# Supplementary material for: Assessing the quality of amoxicillin in the private market in Indonesia: a cross-sectional survey exploring product variety, market volume and price factors
Source: BMJ Open. 2025 Jul 22;15(7):e093785. doi: 10.1136/bmjopen-2024-093785 (PMC12306289; doi:10.1136/bmjopen-2024-093785)
Supplement: online supplemental file 8 [file bmjopen-15-7-s008.pdf]

Supplementary file 8. Estimated market volume of amoxicillin dry syrup products (N=79); sampled, tested and out-of-specification products (source: pharmaceutical analysis & pharmaceutical market data)

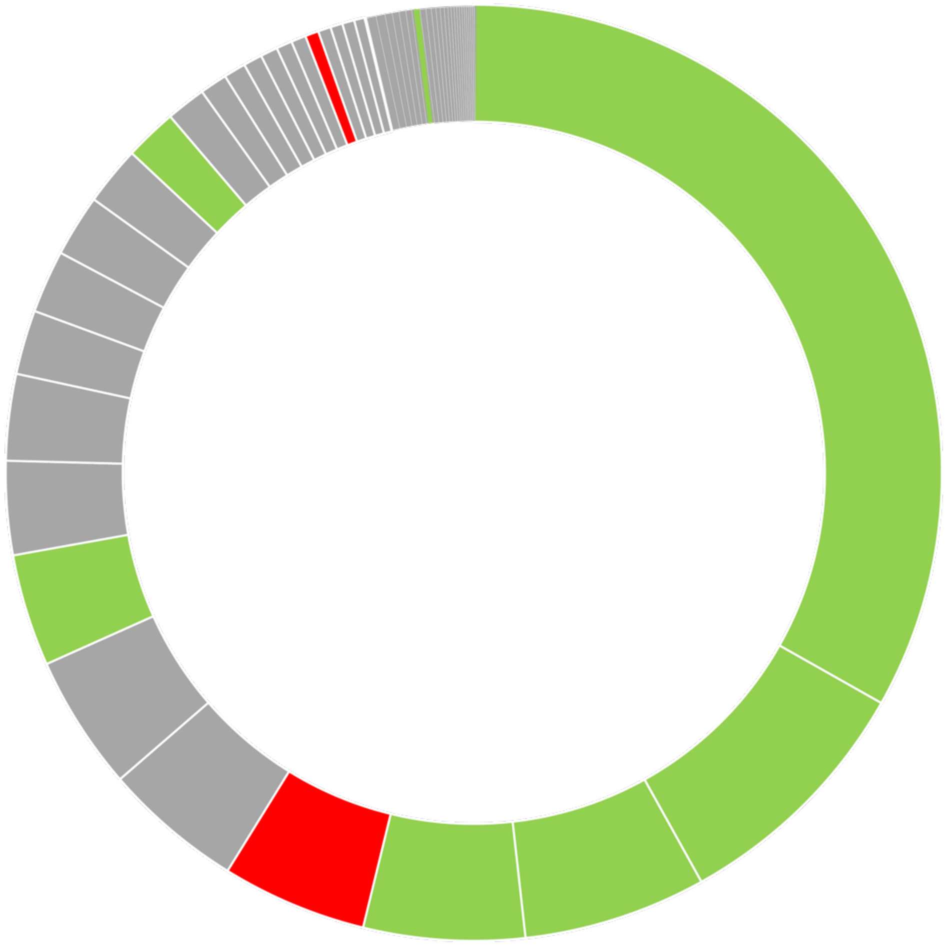

Category	% Estimated market volume
All samples meet specification	65.2%
All samples out-of-specification	8.4%
Not sampled	34.8%

Source: This is based on internal analysis by (Hasnida et al., 2025) using data from the following source: IQVIA MIDAS Quarterly Sales for the period (October 2019-September 2020) reflecting estimates of real-world activity. Copyright IQVIA. All rights reserved.
