# Supplementary material for: Assessing the quality of amoxicillin in the private market in Indonesia: a cross-sectional survey exploring product variety, market volume and price factors
Source: BMJ Open. 2025 Jul 22;15(7):e093785. doi: 10.1136/bmjopen-2024-093785 (PMC12306289; doi:10.1136/bmjopen-2024-093785)
Supplement: Supplementary file 9 [file bmjopen-2024-093785.reviewer_comments.pdf]

PEER REVIEW HISTORY

BMJ Open publishes all reviews undertaken for accepted manuscripts. Reviewers are asked to complete a checklist review form and are provided with free text boxes to elaborate on their assessment. These free text comments are reproduced below.

ARTICLE DETAILS

Title (Provisional)

Assessing the prevalence of poor-quality amoxicillin in the private market in Indonesia: a cross-sectional survey exploring product variety, market volume, and price factors.

Authors

Hasnida, Amalia; Rahmi, Mawadatti; Rahmawati, Ayu; Anggriani, Yusi; van Leth, Frank C.M.; Kok , Maarten Olivier

---

VERSION 1 - REVIEW

**Reviewer**	**1**
**Name**	**Gotham, Dzintars**
**Affiliation**	**Independent**
**Date**	**02-Mar-2025**
**COI**	**None**

Dear Editor and authors,

This is an important study and appears to be rigorous, well written, and transparent. I have minor requests for revisions, mainly relating to contextualization and small wording suggestions.

It was a pleasure to review this study. My congratulations to the authors.

Please see my comments below.

Dzintars Gotham

**

Methods

The term ‘assay’ is used in a very specific jargon way here, which will be confusing for many readers (e.g. where ‘assay’ may be used as synonymous with ‘test’ or ‘diagnostic’). In the Abstract, page 2 lines 45-46, clear definitions of outcomes are given: “the outcomes of pharmaceutical quality testing including the percentage of active pharmaceutical ingredients

(API) and its dissolution." They should be replicated in the main text in Methods, explaining what 'assay' means in this context.

Apologies if I have missed something in the text, but: Why did you not analyse the relationship between assay/dissolution performance and market volume (or market share) based on the MIDAS data?

Data availability statement:

"We are unable to provide the raw, unmasked empirical data of collected samples especially for brand names, name of market authorization holders, and name of manufacturers. Such information might be treated as confidential as it relates to intellectual property."

It is not clear to me why this would be confidential or how it relates to intellectual property. Could the authors explain this or delete the sentence. The point of why the list of specific brands that failed cannot be revealed seems quite key to the policy context. For example, if it is due to a fear of lawfare, that would be important to mention in the limitations of the study.

On page 12 you mention "In accordance with an agreement with the Indonesian medicine regulator, we are not able to report the brands or manufacturers of the tested samples." This statement, which is useful and reasonable, should be copied into the data availability statement.

Results or Discussion:

Table 1 reports 99% domestic manufacture, presumably this refers to the finished pharmaceutical product. Should note also whether the source of API is known (probably not).

Discussion:

While the study focuses on substandard amoxicillin, as far as I can see, the authors found no evidence of falsified amoxicillin products. If correct, that would be important to say clearly in the discussion and abstract. In other words, there is no evidence in this analysis of outright fraud/misrepresentation. Please correct me if wrong.

Page 17 lines 24-27: "Even a slight deviation below the permitted dissolution levels, in particular, supports the development and spread of bacteria resistant to antibiotics[35], a significant concern given the hundreds of millions of amoxicillin dose consumed annually in Indonesia." This statement is quite strong and implies empirical evidence that 'slight deviation' is causing bacterial resistance at the population level. I do not find this statement about a 'slight deviation' supporting the development of resistant bacteria to be supported by reference 35. Could the authors point to where in the cited article (ref 35) this is demonstrated? Alternatively, the sentence could be reworded along the lines of "A low dissolution level could contribute to the development of resistance".

It is striking that only one solid oral formulation product was out of specification in terms of assay percentage (Figure 2). The authors do already point towards this in the Discussion but it could do with even more elucidation: for example, can we conclude that the main challenge – for solid oral forms – is dissolution (and, therefore, issues with formulation of the API / excipients)?

Could the authors suggest some reasons why the dry syrup formulations were out-of-range (much) more frequently than solid oral formulations?

The red vs orange (capsules vs tablets) colour scheme in Figure 2, lower panel, is almost impossible to distinguish. Please change these colours or shapes.

For context, it would be useful in the Discussion if the authors could give an indication of how their study compares to the rest of the literature. Have there been previous similar surveys in Indonesia? Is the present study especially large compared to other previous analyses of SF medicines (my inclination would be yes)? Have 'secret shopper' methods been used many times before? Are there elements of the present study that are methodologically original or improve on previous approaches (I assume this may be the case, and if so, would be worth highlighting).

Small wording suggestions:

Page line 19 "Medicine regulators try to assure…". The wording asserts that medicine regulators are in fact trying (universally). Perhaps more accurate wording would be something like "Medicine regulators are tasked with assuring…".

Page 5 lines 32-33: "An antibiotic like amoxicillin" – you are talking here about amoxicillin, not something like amoxicillin. So I suggest to delete "An antibiotic like".

Page 5 lines 40-41: "After a regulator decides to sample a specific product variation, the difficulty that follows is finding that product in the market.[12]" Please reword – what does 'difficult to find it in the market' mean?

Page 6 line 35: "most widely used antibiotic" …add 'in Indonesia'?

Table one: please correct 'generic' and 'branded' to 'un-branded/INN generic' and 'branded generic', respectively.

**Reviewer**	**2**
**Name**	**Bizimana, Thomas**
**Affiliation**	**University of Rwanda, Pharmacy**
**Date**	**21-Mar-2025**
**COI**	**None**

Review Comments

Title

The title of this article is igeneral and confusing. The title should show that your study was specific to the “Quality and prices of Brands of Amoxicillin formulations found in 4 regions of Indonesia”.

Methods

Study design: Not clear

Population and sample sizes for the medicines outlets: not clear in terms of inclusion criteria and numbers; only the sample definition was provided, and some information should be moved from the “Results” section to make clear the “Methods” section. Where is the randomization recommended by “Reference 39”?

Sampling techniques: with reference to the “sample definition”, on which basis/ calculations did you fix the sample size at 120 samples as your “sampling strategy”? How was this sample representative of the total population/ country’s market volume? How did you capture the aspect of quality variation between different batches? How about the aspect of a batch stored/ found in different regions?

Describe also the methods for the assessment prices. How did you proceed to collect data? What were the reference prices?

Results

For me, this section should start from line 49 of page 13 “Pharmacopeial testing results” and take the preceding sub-sections under “Methods”.

**Reviewer**	**3**
**Name**	**Ashenef, Ayenew**
**Affiliation pharmacongosy**	**Addis Ababa University, Pharmaceutical chemistry and**
**Date**	**21-Mar-2025**
**COI**	**None**

General:

The study attempts to address price, market volume and product variety factors in detecting SF amoxicillin in Indonesian market. It adds value in the medicine quality research theme.

Major:

1. The methodology to calculate market volume was not clear. Please clarify it.

2. Why the selected antibiotic is amoxicillin? Justification should be included in the methodology.

3. Sample definition did not include varieties in batch/lot numbers…if different batch/lot numbers are obtained from single manufacturer in different collection sites how did you treat it? Please clarify the methodology…

4. Dry syrup was included by WHO expert suggestion at the last leg of your study? What is the impact on the overall conclusion and study limitation? Please discuss it explicitly.

5. Sampling strategy was aimed at 120 samples. By what formulae you come to the mentioned figure…Please explain it in the manuscript.

6. Why the sampled amoxicillin was produced domestically only? Is there a closed market system in Indonesia?

7. In figure 1..Dissolution round tests total only 53(the passed samples N value) rather than 57. Why the distortion?

8. Figure 2 : Market share figure is not clear to comprehend it.

9. The samples information, the brands, names, batch/lot numbers, strengths, manufacturing date, expiry date etc……should be included as raw data supplementary file

10. The analytical results in terms of assay, HPLC chromatogrammes, dissolution data should be included as supplementary for each sample tested. The comment sin 9 and 10 are in line with open access publication policies….

Minor;

1. Typos, grammatical and writing errors in some points should be addressed (e.g. Reference numbers after the dot etc…)

2. Although ethical clearance was obtained in Indonesia…Why an additional ethical clearance was not obtained from the Netherlands where the researchers are affiliated with?

The Manuscript is a good scientific compilation and work thus adds value to medicine quality studies and after addressing the above comments, I recommend publication in BMJ open…………

VERSION 1 - AUTHOR RESPONSE

Reviewer 1

Dear Editor and authors,
This is an important study and appears to be rigorous, well written, and transparent. I have minor requests for revisions, mainly relating to contextualization and small wording suggestions.
It was a pleasure to review this study. My congratulations to the authors.
Please see my comments below.

Dzintars Gotham

We thank Reviewer 1 for taking the time to review the manuscript and for the valuable insights, kind words, and constructive suggestions.. Below, we address these suggestions one by one and indicate the changes that we made in the manuscript. These are highlighted in green.

Methods

The term ‘assay’ is used in a very specific jargon way here, which will be confusing for many readers (e.g. where ‘assay’ may be used as synonymous with ‘test’ or ‘diagnostic’). In the Abstract, page 2 lines 45-46, clear definitions of outcomes are given: “the outcomes of pharmaceutical quality testing including the percentage of active pharmaceutical ingredients (API) and its dissolution.” They should be replicated in the main text in Methods, explaining what ‘assay’ means in this context.
Thank you for point this out. We have added specific definitions of assay and dissolution in the context of medicine quality testing to the Methods (page 10, lines 254-256).

Apologies if I have missed something in the text, but: Why did you not analyse the relationship between assay/dissolution performance and market volume (or market share) based on the MIDAS data? Thank you for your suggestion. We added this analysis and found no relationship between the estimated market volume of oral solid products and quality testing. We mentioned this in the Methods (page 11, lines 303-306) and the Results (page 16, lines 420-422).

Data availability statement:
“We are unable to provide the raw, unmasked empirical data of collected samples especially for brand names, name of market authorization holders, and name of manufacturers. Such information might be treated as confidential as it relates to intellectual property.”
It is not clear to me why this would be confidential or how it relates to intellectual property. Could the authors explain this or delete the sentence. The point of why the list of specific brands that failed cannot be revealed seems quite key to the policy context. For example, if it is due to a fear of lawfare, that would be important to **mention in the limitations** of the study. Thank you for this comment. We have added more detail to the Data Availability Statement (page 22, lines 615–623). Due to our agreement with the Indonesian Medicines Regulator, we are unable to share brand names, manufacturer names, and the names of market authorization holders. We do not consider this a limitation of the study, as it does not affect the results or the interpretation of the findings. These data were shared with the regulator, who is responsible for taking follow-up actions with the manufacturers.

On page 12 you mention “In accordance with an agreement with the Indonesian medicine regulator, we are not able to report the brands or manufacturers of the tested samples.” This statement, which is useful and reasonable, should be copied into the data availability statement. We have copied the suggested statement into the Data Availability Statement (page 22, lines 617-619).

Results

Table 1 reports 99% domestic manufacture, presumably this refers to the finished pharmaceutical product. Should note also whether the source of API is known (probably not). We clarified that we collected samples as finished pharmaceutical products (page 13 line 354), and we had no information on the source of APIs of each sample (page 13 lines 360-361).

Discussion

While the study focuses on substandard amoxicillin, as far as I can see, the authors found no evidence of falsified amoxicillin products. If correct, that would be important to say clearly in the discussion and abstract. In other words, there is no evidence in this analysis of outright fraud/misrepresentation. Please correct me if wrong. Thank you for raising this point. While several samples tested out of specification, all products contained the stated active ingredient, and the packaging appeared normal. However, we cannot claim that there was no evidence of falsified amoxicillin products, as we did not have the means or authority to verify the authenticity of the collected samples. We have added this clarification in the Results section (page 14, lines 373-374) and Discussion (page 18, lines 453-457).

Page 17 lines 24-27: "Even a slight deviation below the permitted dissolution levels, in particular, supports the development and spread of bacteria resistant to antibiotics[35], a significant concern given the hundreds of millions of amoxicillin dose consumed annually in Indonesia." This statement is quite strong and implies empirical evidence that 'slight deviation' is causing bacterial resistance at the population level. I do not find this statement about a 'slight deviation' supporting the development of resistant bacteria to be supported by reference 35. Could the authors point to where in the cited article (ref 35) this is demonstrated? Alternatively, the sentence could be reworded along the lines of "A low dissolution level could contribute to the development of resistance". Thank you for this suggestion. We have followed the suggestion to reword the text and have replaced the reference with a more appropriate source in the Discussion section (page 18, lines 462-463).

It is striking that only one solid oral formulation product was out of specification in terms of assay percentage (Figure 2). The authors do already point towards this in the Discussion but it could do with even more elucidation: for example, can we conclude that the main challenge – for solid oral forms – is dissolution (and, therefore, issues with formulation of the API / excipients)? Thank you for raising this question. We agree with the interpretation of the reviewer. We do believe this finding needs further study by pharmaceutical scientists. We have mentioned this point in the Discussion (page 18, lines 476-479).

Could the authors suggest some reasons why the dry syrup formulations were out-of-range (much) more frequently than solid oral formulations? Thank you for your suggestion. With the data that we have, we are not able to suggest reasons for the poor quality of the dry syrup formulations. We do believe this requires further study by pharmaceutical scientists or chemists. We have added this suggestion for further study in the Discussion (page 18, lines 478-481).

The red vs orange (capsules vs tablets) colour scheme in Figure 2, lower panel, is almost impossible to distinguish. Please change these colours or shapes. Thank you for pointing this out. We have changed the colour and added more detail to differentiate the shapes of capsules and tablets in Figure 3.

For context, it would be useful in the Discussion if the authors could give an indication of how their study compares to the rest of the literature. Have there been previous similar surveys in Indonesia? We have explicitly mentioned previous similar surveys in Indonesia in the Discussion (page 19, lines 482-483).

Is the present study especially large compared to other previous analyses of SF medicines (my inclination would be yes)? What is especially large in this study compared to previous studies of SF medicines is the product diversity. We have underlined this point in the Discussion (page 19, lines 480-483) and the Strengths and Limitations (pages 20-21, lines 551-554).

Have 'secret shopper' methods been used many times before? Mystery shoppers have been suggested as an effective approach to collecting or purchasing samples, and they have been frequently used in previous surveys of SF medicines. We have explicitly added this point in the Discussion (page 20, lines 532-534).

Are there elements of the present study that are methodologically original or improve on previous approaches (I assume this may be the case, and if so, would it be worth highlighting)? Thank you for this suggestion. Our study includes two methodological advances: product diversity sampling and the incorporation of market volume in our analysis. We have expanded the discussion of these in the Discussion section (page 19, lines 480-483 and lines 490-493).

Small wording suggestions

Page line 19 "Medicine regulators try to assure…". The wording asserts that medicine regulators are in fact trying (universally). Perhaps more accurate wording would be something like "Medicine regulators are tasked with assuring…".
Page 5 lines 32-33: "An antibiotic like amoxicillin" – you are talking here about amoxicillin, not something like amoxicillin. So I suggest to delete "An antibiotic like".
Page 5 lines 40-41: "After a regulator decides to sample a specific product variation, the difficulty that follows is finding that product in the market.[12]" Please reword – what does 'difficult to find it in the market' mean?
Page 6 line 35: "most widely used antibiotic" …add 'in Indonesia'?
Table one: please correct 'generic' and 'branded' to 'un-branded/INN generic' and 'branded generic', respectively.

Thank you for these wording suggestions, which we all incorporated into the text.

Reviewer 2

We thank Reviewer 2 for taking the time to review our article and provide constructive suggestions that helped us improve our article. Below, we address these suggestions one by one and indicate the changes that we made in the manuscript. These are highlighted in green.

Title

The title of this article is igeneral and confusing. The title should show that your study was specific to the "Quality and prices of Brands of Amoxicillin formulations found in 4 regions of Indonesia". Thank you for the suggestion regarding our title. We carefully considered it and discussed it with our colleagues. However, we prefer to retain the current title, as we believe that price and market volume are central aspects of our study and should be reflected in the title. Additionally, our focus is on products, not brands. In the Indonesian market, multiple products with different dosage forms and strengths can exist under the same brand name. Therefore, we refer to "amoxicillin products" rather than "amoxicillin brands." We have tried to better explain this in the text, including in our sampling definition in the Methods (page 6, lines 152-156).

Methods

Study design: Not clear. Thank you for pointing this out. We have specifically stated our study design at the beginning of the Methods section (page 6, lines 141-142).

Population and sample sizes for the medicines outlets: not clear in terms of inclusion criteria and numbers; only the sample definition was provided, and some information should be moved from the "Results" section to make clear the "Methods" section. Thank you for pointing this out. We tried to write it down more clearly in the Methods (page 7, lines 197-205). Since testing medicine in the laboratory can be quite expensive (Valente et al., 2024; doi:10.1136/bmjgh2024-015402), we determined the maximum sample size given the financial budget. We anticipated that our sample size (n=120) would cover around 90% of the estimated market volume share.

Where is the randomization recommended by "Reference 39"? In our study, we specifically chose a purposive sampling strategy focusing on product variety, as we underlined in the Methods (page 7, lines 200-203). We chose for a purposive sampling strategy because there is a small number of products that have a large market share, and there are many more products with a small market share. A random sampling strategy would most likely resulted in sampling many of the same products. By following a purposive sampling strategy, we have most likely achieved a more diverse sample, with a larger number of different products.

Sampling techniques: with reference to the "sample definition", on which basis/ calculations did you fix the sample size at 120 samples as your "sampling strategy"? Thank you for this question. The essence of our purposive sampling strategy was to aim for the largest possible variety of products that we could find in the four sampling regions and online. The total sample size (N=120) is based upon the maximum feasible budget. We have added this in the Methods (page 7, lines 203-205).

How was this sample representative of the total population/ country's market volume? Thank you for this question. Our study focuses only on the products available in private outlets such

as private pharmacies and drug stores. Since we did not sample products in the public section, our study does not claim to be representative of the total population / the country's market volume. We have included this remark in the Discussion (page 19, lines 513-514) and clarified the points again in the Strengths and Limitations (pages 21, lines 554-559).

How did you capture the aspect of quality variation between different batches? Thank you for this question. In our study, we focused on different products, defined in Table 1 in the Results as "one amoxicillin brand, of one dosage, in tablets or capsules or dry syrups, and from one manufacturer". As such, we assessed the quality of different products and not different batches. In Table 1, we do report the samples that came from more than one batch number, which is 7.5%. We believe that a different study design and a sampling strategy is required to effectively assess the quality variation between different batches of the same product. In the Discussion, we recommend an analysis of batch-to-batch variation for further research (page 19, lines 499-501).

How about the aspect of a batch stored/ found in different regions? This is, again, a very interesting and relevant question, but our focus in this study was on products and not batches. Collecting samples from different batches and testing their quality would require a different sampling strategy and a much larger sample size.

Describe also the methods for the assessment prices. How did you proceed to collect data? What were the reference prices? Thank you for this suggestion. We collected prices through mystery shoppers. We do not refer to any reference prices, as we focus on the prices that are paid by patients/consumers. We have added this information in the Methods (page 11, lines 287-291) and clarified the analysed price in the Results (page 17, lines 430).

Results

For me, this section should start from line 49 of page 13 "Pharmacopeial testing results" and take the preceding sub-sections under "Methods". We understand this suggestion, but we believe that the order in which we present the Results section is appropriate since we follow a product variety sampling strategy. In the Methods section, we lay out our sampling strategy (pages 7-8, lines 197-211). In the Results section, we present the product variety that we identified in the field, which we believe is a first result, and a part of our sampling outcomes (page 12, lines 335-351). We continue with the description of the collected samples (page 13, lines 353-363), which followed by quality testing results (pages 14-16, lines 372-426).

Thanks a lot for the review.

Reviewer 3

General:
The study attempts to address price, market volume and product variety factors in detecting SF amoxicillin in Indonesian market. It adds value in the medicine quality research theme.

We thank Reviewer 3 for taking the time to review the manuscript and for the valuable insights and constructive suggestions for improvement. Below, we address these suggestions

one by one and indicate the changes that we made in the manuscript. These are highlighted in green.

Major:

1. The methodology to calculate market volume was not clear. Please clarify it. Thank you for this question. We obtained the estimated market volume data from the commercial data provider. This is the MIDAS data that is available from the company named IQVIA. We have clarified this in Methods (page 7, lines 185-188).

2. Why is amoxicillin the selected antibiotic? Justification should be included in the methodology. Thank you for your suggestion. We briefly mentioned our justification for selecting amoxicillin in the Introduction (page 5, lines 118-120) and described it in more detail in the Methods (page 6, lines 143-150).

3. Sample definition did not include varieties in batch/lot numbers…if different batch/lot numbers are obtained from single manufacturer in different collection sites how did you treat it? Please clarify the methodology… Thank you for raising this. Our definition of a sample is "one finished pharmaceutical product (with amoxicillin as active pharmaceutical ingredient/API, of one dosage (strength and form), of one brand, from one manufacturer, and collected at one location, at one time". As the reviewer points out, our definition does not include batch numbers because our study focused on products as the unit of analysis. In Table 1, we do provide information about the number of samples that have the same batch number (92.5%). We believe that comparing the quality of product with different batch numbers from a single manufacturer in different collection sites would require a different sampling strategy and a much larger sample, which is beyond the scope of our study. In the Discussion, we suggest that further research could look into batch-to-batch variation (page 19, lines 499-501).

4. Dry syrup was included by WHO expert suggestion at the last leg of your study? What is the impact on the overall conclusion and study limitation? Please discuss it explicitly. Thank you for your suggestion. Dry syrups were only sampled in our last sampling location in Greater Jakarta and seemed more likely to test out-of-specification in the assay. We have added more details about this in Table 2, separating the location data for each formulation type and pharmaceutical analysis results. We also add a remark about this in the Results (page 15, lines 381-383 and page 15, lines 399-401) and limitations in the Discussion (page 21, lines 557-559).

5. Sampling strategy was aimed at 120 samples. By what formulae you come to the mentioned figure…Please explain it in the manuscript. We aim to sample the largest possible product variety in these selected sampling regions. The total sample size is the maximum we could afford in our budget. We explained this in detail in the Methods (page 7, lines 197-205).

6. Why the sampled amoxicillin was produced domestically only? Is there a closed market system in Indonesia? Thank you for this question. Indonesia has a large domestic pharmaceutical manufacturing that aims to sufficiently meet national demand. While Indonesia has an open market system, including for imported products, amoxicillin

manufacturing is dominated by domestic producers. We added this point in the Discussion (page 19, lines 488-491).

7. In figure 1..Dissolution round tests total only 53(the passed samples N value) rather than 57. Why the distortion? Thank you for this question. There were, in total, four samples that were ineligible for dissolution round 2. At the start of dissolution testing round 2, three samples exceeded the expiration date. For one sample, we did not have the required minimum quantity of 40 tablets. We have clarified this in the Results (page 15, lines 382-385).

8. Figure 2: The market share figure is not clear. We adjusted Figure 2 to make it easier to comprehend. We have replaced the doughnut chart, with a treemap chart that shows the market volume of all authorized Amoxicillin products.

9. The samples information, the brands, names, batch/lot numbers, strengths, manufacturing date, expiry date etc…...should be included as raw data supplementary file. For regulatory purposes, we provided this information to the Indonesian medicine regulator. We are not allowed to
provide these details (brands, names, manufactures, batch/lot numbers) of the samples because we are bound by the agreement with the regulator. We have pointed out these considerations in the Results (page 13, lines 362-363) and Data Availability Statement (page 22, lines 617-619). As we point out in our data availability statement, we welcome other researchers to contact us for the masked raw data.

10. The analytical results including assay, HPLC chromatograms, and dissolution data, should be included as supplementary information for each sample tested. The comments in 9 and 10 are in line with open access publication policies….
After the publication of the article, we are allowed to share the masked analytical results, as stated in the Data Availability Statement (page 22, lines 621-623). We welcome other researchers to contact us for the masked raw data.

Minor

Minor;

1. Typos, grammatical and writing errors in some points should be addressed (e.g. Reference numbers after the dot, etc…). Thank you for raising this point. We have made adjustments as necessary, including updating the reference number.

2. Although ethical clearance was obtained in Indonesia…Why an additional ethical clearance was not obtained from the Netherlands where the researchers are affiliated with? Our team is predominantly comprised of researchers of Indonesian nationality, and most of our study team is based in Indonesian institutions. Our empirical study was conducted in Indonesia. We obtained ethical clearance from the Indonesian Institutional Review Board (IRB) in line with national policies and regulations. We believe that local ethical clearance is of utmost importance to ensure that good research practices will be followed throughout this study's planning, implementation, and reporting at the local level.

The Manuscript is a good scientific compilation and work thus adds value to medicine quality studies and after addressing the above comments, I recommend publication in BMJ open…………

We thank Reviewer 3 for the review and constructive suggestions.

VERSION 2 - REVIEW

**Reviewer**	**1**
**Name**	**Gotham, Dzintars**
**Affiliation**	**Independent**
**Date**	**25-May-2025**
**COI**	

Thank you, all comments have been addressed, except one. The point that remains to be addressed is regarding whether or not the authors found any evidence of falsification.

As far as I can see, no evidence was found. Additionally, the study was of course 'powered' to give good coverage of the market. Therefore, the study should say "This analysis did not find any evidence of falsification." or some similar wording, which is an accurate reporting of the findings.

The revised manuscript currently reads:

"Although we did not find any samples which were entirely depleted of amoxicillin or contained a different API, we could not assert that we have not found falsified products since we had no means and authority to perform the authenticity check of the products as usually performed by the medicine regulator."

I do not understand why the authors 'cannot assert that they have not found falsified products'. If no evidence is found for something, it is standard to state so. If an observational study finds no evidence of a correlation between HbA1c and long-term mortality, then the study would state that its analysis did not find evidence of a correlation.

I am also not sure why the authors assert they did not have the 'means' of checking authenticity in "as usually performed by the medicine regulator." Does the regulator use different analytical methods? Are they superior to the methods used by the authors? This should be explained or the sentence should be removed.

If the authors feel it is important to say that they are not 100% certain that no falsified products exist in Indonesia, they should say this in a separate statement. For example, the relevant section could be expressed as follows:

"We did not find any samples which suggested a falsified medicine (e.g. samples entirely lacking amoxicillin or containing a different API). We cannot assert with confidence that no falsified amoxicillin exists in Indonesia and emphasize that monitoring for falsified products is the role of the national medicine regulator."

This aspect does need to be addressed before publication.

Thank you again for the opportunity to review this interesting and valuable manuscript.

Dzintars Gotham

**Reviewer**	**3**
**Name**	**Ashenef, Ayenew**
**Affiliation**	**Addis Ababa University, Pharmaceutical chemistry and pharmacongosy**
**Date**	**30-May-2025**
**COI**	

The Authors Had addressed all of my comments in per my previous suggestions. Thus, I recommend publication of this manuscript.

VERSION 2 - AUTHOR RESPONSE

Reviewer 1

Dr. Dzintars Gotham, Independent
Comments to the Author:
Thank you, all comments have been addressed, except one. The point that remains to be addressed is regarding whether or not the authors found any evidence of falsification.
As far as I can see, no evidence was found. Additionally, the study was of course 'powered' to give good coverage of the market. Therefore, the study should say "This analysis did not find any evidence of falsification." or some similar wording, which is an accurate reporting of the findings.

The revised manuscript currently reads:
"Although we did not find any samples which were entirely depleted of amoxicillin or contained a different API, we could not assert that we have not found falsified products since we had no means and authority to perform the authenticity check of the products as usually performed by the medicine regulator."

I do not understand why the authors 'cannot assert that they have not found falsified products'. If no evidence is found for something, it is standard to state so. If an observational study finds no evidence of a correlation between HbA1c and long-term mortality, then the study would state that its analysis did not find evidence of a correlation.

I am also not sure why the authors assert they did not have the 'means' of checking

authenticity in "as usually performed by the medicine regulator." Does the regulator use different analytical methods? Are they superior to the methods used by the authors? This should be explained or the sentence should be removed.

Thank you for this question. We agree with Reviewer 1 and have simplified that text in the manuscript (page 18, lines 453-455).

If the authors feel it is important to say that they are not 100% certain that no falsified products exist in Indonesia, they should say this in a separate statement. For example, the relevant section could be expressed as follows:

"We did not find any samples which suggested a falsified medicine (e.g. samples entirely lacking amoxicillin or containing a different API). We cannot conclude that no falsified amoxicillin exists in Indonesia and emphasize that monitoring for falsified products is the role of the national medicine regulator."

This aspect does need to be addressed before publication.

Thank you for this suggestion. We have incorporated the following sentences in the manuscript (page 18, lines 453-455).

We did not identify any samples indicative of falsified medicines (e.g., those completely lacking amoxicillin or containing a different active pharmaceutical ingredient).

Thank you again for the opportunity to review this interesting and valuable manuscript.
Dzintars Gotham

We thank Reviewer 1 for the valuable input to improve our manuscript.

Reviewer 3

Reviewer: 3
Dr. Ayenew Ashenef, Addis Ababa University, Center for Innovative Drug Development and Therapeutic Trials for Africa (CDT-Africa)
Comments to the Author:
The Authors Had addressed all of my comments in per my previous suggestions. Thus, I recommend publication of this manuscript.

We thank Reviewer 3 for reviewing our manuscript and recommend its publication.
